# Trichome regulator SlMIXTA‐like directly manipulates primary metabolism in tomato fruit

**DOI:** 10.1111/pbi.13202

**Published:** 2019-07-24

**Authors:** Shiyu Ying, Min Su, Yu Wu, Lu Zhou, Rao Fu, Yan Li, Hao Guo, Jie Luo, Shouchuang Wang, Yang Zhang

**Affiliations:** ^1^ Key Laboratory of Bio‐resource and Eco‐environment of Ministry of Education College of Life Sciences State Key Laboratory of Hydraulics and Mountain River Engineering Sichuan University Chengdu China; ^2^ National Key Laboratory of Crop Genetic Improvement and National Center of Plant Gene Research (Wuhan) Huazhong Agricultural University Wuhan China; ^3^ Hainan Key Laboratory for Sustainable Utilisation of Tropical Bioresource College of Tropical Crops Hainan University Haikou China

**Keywords:** tomato, mGWAS, SlMIXTA‐like, primary metabolism, secondary metabolism, phenylpropanoid, 3‐Deoxy‐d‐arabinoheptulosonate 7‐phosphate synthase

## Abstract

Trichomes are storage compartments for specialized metabolites in many plant species. In trichome, plant primary metabolism is significantly changed, providing substrates for downstream secondary metabolism. However, little is known of how plants coordinate trichome formation and primary metabolism regulation. In this report, tomato (*Solanum lycopersicum*) trichome regulator *SlMIXTA‐like* is indicated as a metabolic regulation gene by mGWAS analysis. Overexpression of *SlMIXTA‐like* in tomato fruit enhances trichome formation. In addition, SlMIXTA‐like can directly bind to the promoter region of gene encoding 3‐deoxy‐7‐phosphoheptulonate synthase (*SlDAHPS*) to activate its expression. Induction of *SlDAHPS* expression enhances shikimate pathway activities and provides substrates for downstream secondary metabolism. Our data provide direct evidence that trichome regulator can directly manipulate primary metabolism, in which way plants can coordinate metabolic regulation and the formation of storage compartments for specialized metabolites. The newly identified *SlMIXTA‐like* can be used for future metabolic engineering.

## Introduction

Phenylpropanoid compounds are ubiquitous in plant kingdom. They are associated with almost all important physiological processes: from the formation of plant architecture to stress response, as well as plant reproduction and symbiosis (Vogt, 2010). In addition, the health benefits of phenylpropanoids have been extensively investigated during the past 20 years. Many studies have provided evidences that phenylpropanoid compounds play positive roles in human health such as chronic disease preventing, anticancer and anti‐ageing (Butelli *et al*., 2008; Carmona‐Gutierrez *et al*., 2019; Martin and Li, 2017; Scarano *et al*., 2017). As a result, phenylpropanoids with health benefits are often the main targets of metabolic engineering. During the past decade, the development of tools and experiences enable us to engineer various secondary metabolites in plant systems (Patron *et al*., 2015). Among popular plant production platforms, tomato is one of the most desirable chassis, due to its high yield and short life cycle, as well as its capability to apply most modern biotechnologies (Li *et al*., 2018).

One common strategy to improve the efficiency of metabolic engineering in plant chassis is the application of transcription factors (TFs) (Century *et al*., 2008; Fu *et al*., 2018). Previous studies indicate MYB proteins are main regulators for phenylpropanoid metabolism and MYBs with activation function are found to mainly belong to subgroups 5, 6, 7 and 27 (Liu *et al*., 2015). Fruit‐specific expression of these TFs can significantly improve the production of valuable metabolites in tomato fruit (Bovy *et al*., 2002; Butelli *et al*., 2008; Gonzali *et al*., 2009; Luo *et al*., 2008).

It was not until recently did we begin to realize the importance of primary metabolism regulation in metabolic engineering (Morandini, 2013). Phenylpropanoids are synthesized from phenylalanine, which are the main products of shikimate pathway (Vogt, 2010). Phosphoenolpyruvate from glycolysis and erythrose‐4‐phosphate from pentose phosphate pathway are the substrates for the first step of shikimate pathway, and 3‐Deoxy‐d‐arabinoheptulosonate 7‐phosphate synthase (DAHPS) catalysed this reaction. Previously indicated overexpression of *DAHPS* in plants can significantly enhance the activities of shikimate pathway, leading the metabolic flux towards phenylpropanoids biosynthesis (Tzin *et al*., 2012, 2013). Coincidentally, overexpression of *AtMYB12*, a flavonol regulator in *Arabidopsis thaliana*, in tomato fruit was found to significantly enhance the expression of genes involved in glycolysis, pentose phosphate pathway and shikimate pathway (Fu *et al*., 2018; Luo *et al*., 2008; Zhang *et al*., 2015). Further investigation indicates that AtMYB12 directly activates the expression of genes encoding DAHPS and enolase (ENO). And activation of these two genes redirects the carbon flux towards aromatic amino acid biosynthesis, providing substrates for downstream secondary metabolic pathways. (Fu *et al*., 2018; Luo *et al*., 2008; Zhang *et al*., 2015). All these indicate that reprogramme of primary metabolism can be achieved by manipulating the activities of several key enzymes. And some specific TFs can regulate the expression of genes encoding these enzymes therefore guide the carbon flux towards certain pathways.

Based on this theory, it is possible to design the plant carbon flux once regulatory mechanism for key genes in primary metabolism been identified (Tzin *et al*., 2012, 2013). And there are examples that a single TF can activate those key genes (Zhang *et al*., 2015). In addition to pathway reconstruction, using TFs to control both primary and secondary metabolic pathways has become a new generation of methodology in plant metabolic engineering (Fu *et al*., 2018). As a result, the discovery and identification of new TFs are vital for successful metabolic engineering. So far, the most successful strategies are using natural mutants and through mutagenesis approaches (Adato *et al*., 2009; Ballester *et al*., 2009; Borevitz *et al*., 2000; Schwinn *et al*., 2006). Recently, multi‐omics integration analysis was used to link genetic basis and metabolic changes in tomato breeding. The genome, transcriptome and metabolome of 610 tomato germplasms revealed the molecular basis for the domestication of important agronomic traits in tomato (Zhu *et al*., 2018). In addition to its importance in studying tomato domestication process, this data set is a vital resource for dissecting plant metabolic regulation in high throughput.

Compared to microbial system, one unique character of plant chassis is that the production of metabolites is tissue‐specific. Trichomes are common structures for plants to store specialized metabolites, particularly chemicals involved in stress and disease tolerance (Glas *et al*., 2012; Huchelmann *et al*., 2017). In order to produce large amount of metabolites, carbon flux has been significantly changed in trichomes (Balcke *et al*., 2017). Therefore, the plants may have a regulatory mechanism to coordinate primary metabolism regulation and trichome formation. Previous studies indicate MIXTA‐like MYB TFs are responsible for trichome formation in many plants (Glover *et al*., 1998; Lashbrooke *et al*., 2015; Perez‐Rodriguez *et al*., 2005). And recent study in *Artemisia annua* indicates trichome and artemisinin regulator 1 (TAR1), an AP2 transcription factor, is required for both trichome development and artemisinin biosynthesis (Tan *et al*., 2015). However, how can plant significantly reprogramme the primary metabolism in trichome is still unknown. In this study, we performed mGWAS for a recently published tomato multi‐omics data set (Zhu *et al*., 2018). We identified *SlMIXTA‐like* (*Solyc02g088190*), a gene encoding a MIXTA‐like TF, which belongs to subgroup 9 MYB family, as a direct regulator of tomato primary metabolism. Our finding provides evidence that trichome regulator has direct roles in primary metabolic regulation and this can be used for future metabolic engineering.

## Results

### *SlMIXTA‐like* is the candidate gene for a major phenylpropanoid QTN

**Figure 1:**
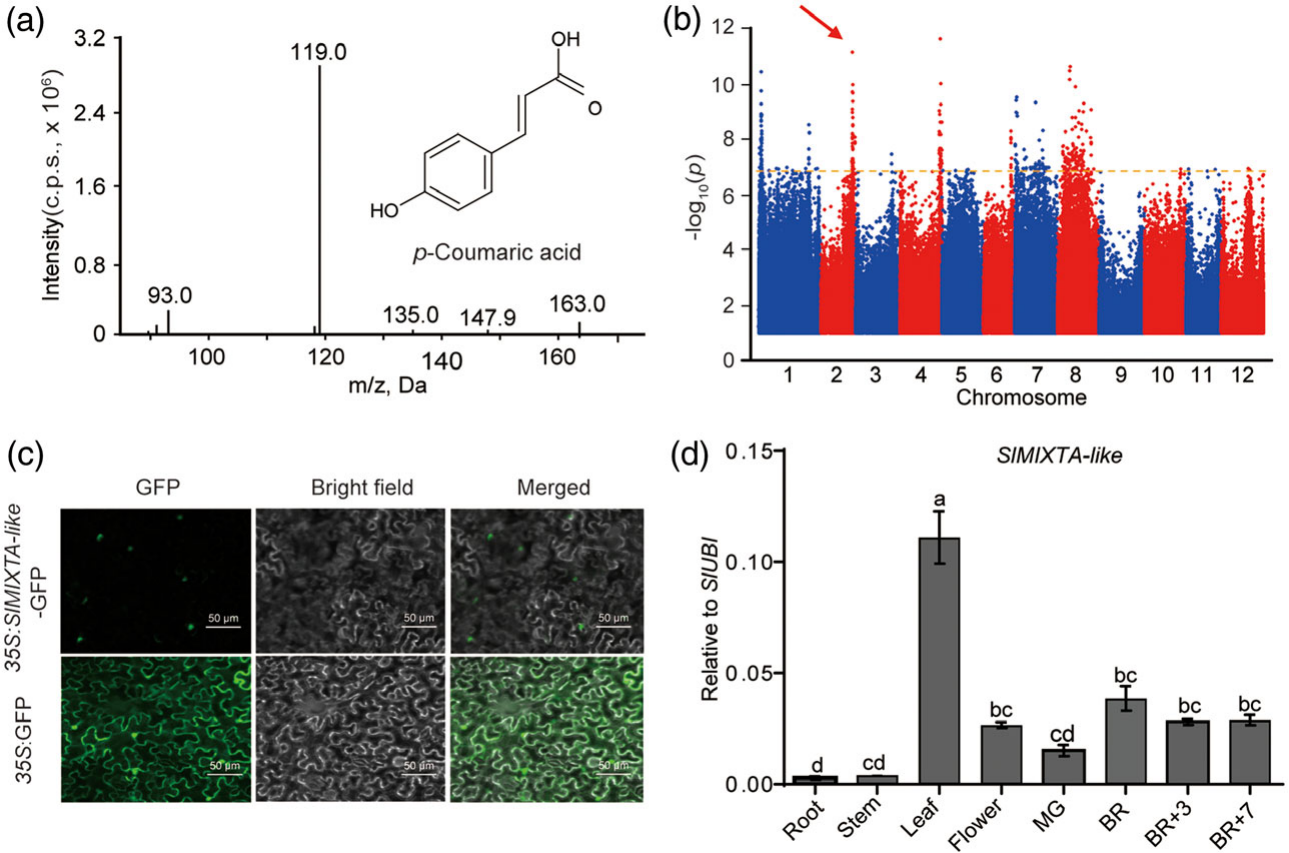
*SlMIXTA‐like* is the candidate gene for a major phenylpropanoid QTN. (a) Structure and MS/MS profile of compound SlFM0124, ρ‐coumaric acid. (b) Manhattan plot for the metabolite of SlFM0124, ρ‐coumaric acid. A significant QTN was shown on chromosome 2. (c) SlMIXTA‐like is a nuclei localized protein. Full‐length *SlMIXTA‐like* cDNA was fused with GFP. Agroinfiltrated *Nicotiana benthamiana* leaves were analysed at 3dpi. (d) RT‐qPCR data indicate tissue‐specific expression pattern of *SlMIXTA‐like* in MicroTom. Error bars show SEM (*n* = 3). Different letters indicate significantly different values at *P* < 0.05 (one‐way ANOVA, Tukey's *post hoc* test).

In order to screen new TFs regulating important metabolites in tomato, we checked through the tomato multi‐omics data set from our co‐authors (see Experimental procedures) (Zhu *et al*., 2018). We noticed a significant quantitative trait nucleotide (QTN) (*P* = 1.93E‐10) between the levels of an important phenylpropanoid compound—ρ‐coumaric acid (SlFM0124) and a SNP (sf0250278120) on chromosome 2 (Figure 1a, b). This SNP is located 74 Kb away from gene *Solyc02g088190* (Data S1). Using haplotype analysis, we found that the two SNPs in the CDS region of *Solyc02g088190* gene have significant correlation with the content of ρ‐coumaric acid (sf0250353631 and sf0250353789; *P* = 2.17E‐16 and 4.71E‐18, respectively) (Figure S1), Molecular phylogenetic analysis of the R2R3‐MYB transcription factors from *Arabidopsis thaliana* and tomato revealed that the Solyc02g088190 belongs to the subgroup 9 (Figure S2 and S3) (Liu *et al*., 2015). It has previously been reported as *SlMIXTA‐like*, a MIXTA‐like R2R3 MYB TF, which was found to be linked to conical cell development and trichome formation (Ewas *et al*., 2016; Lashbrooke *et al*., 2015). As trichomes have been shown to produce and store various metabolites, phenylpropanoids, included, we predict *SlMIXTA‐like* is the candidate gene underlining this locus.

As previously described, SlMIXTA‐like is a nucleus‐localized protein (Figure 1c) (Dubos *et al*., 2010; Ewas *et al*., 2016; Lashbrooke *et al*., 2015). To better investigate the role of *SlMIXTA‐like*, we analysed *SlMIXTA‐like* expression pattern in different tissues using RT‐qPCR. The transcript level of *SlMIXTA‐like* reaches its highest in leaves while remains a relative high level in flower and different fruit stages (Figure 1d). Similar result has also been shown from TomExpress website (Figure S4) (Zouine *et al*., 2017).

**Figure 2:**
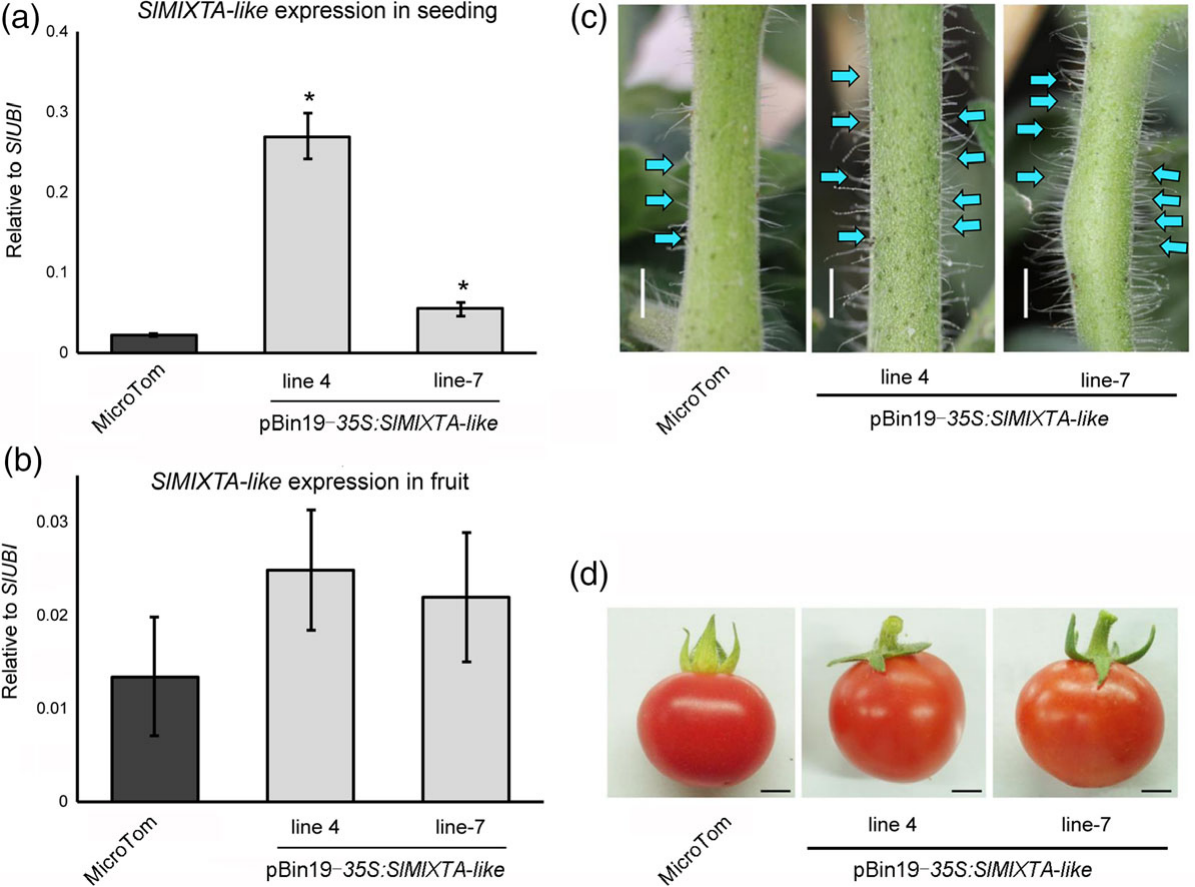
Constitutive expression of *SlMIXTA‐like* in tomato plant enhances trichome formation in seedlings. (a) Expression levels of *SlMIXTA‐like* in MicroTom and *35S:SlMIXTA‐like* seedlings. (b) Enhanced trichome formation on the stem of *35S:SlMIXTA‐like* seedlings. Scale bars show 4 mm. (c) *SlMIXTA‐like* expression levels in the fruit of MicroTom and *35S:SlMIXTA‐like* plants. (d) Phenotype of ripe fruit of MicroTom and *35S:SlMIXTA‐like*. Scale bars show 4 mm. *P < 0.05.

In order to verify *SlMIXTA‐like*'s function, we first overexpressed *SlMIXTA‐like* under CaMV *35S* promoter. Compared to MicroTom, the *SlMIXTA‐like* expression levels are significantly increased in the seedlings of transgenic lines (Figure 2a). As previously reported, enhanced trichome formation was observed in the stem of transgenic lines (Figure 2c and Figure S5). To analyse effects of *SlMIXTA‐like* overexpression in tomato fruit, we then checked the expression level of *SlMIXTA‐like* in transgenic fruit. However, due to the limitation of *35S* promoter, the expression of *SlMIXTA‐like* was very low in transgenic fruit and no significant phenotypic changes were observed (Figure 2b, d).

### Fruit‐specific expression of *SlMIXTA‐like* significantly changes fruit phenotype

**Figure 3:**
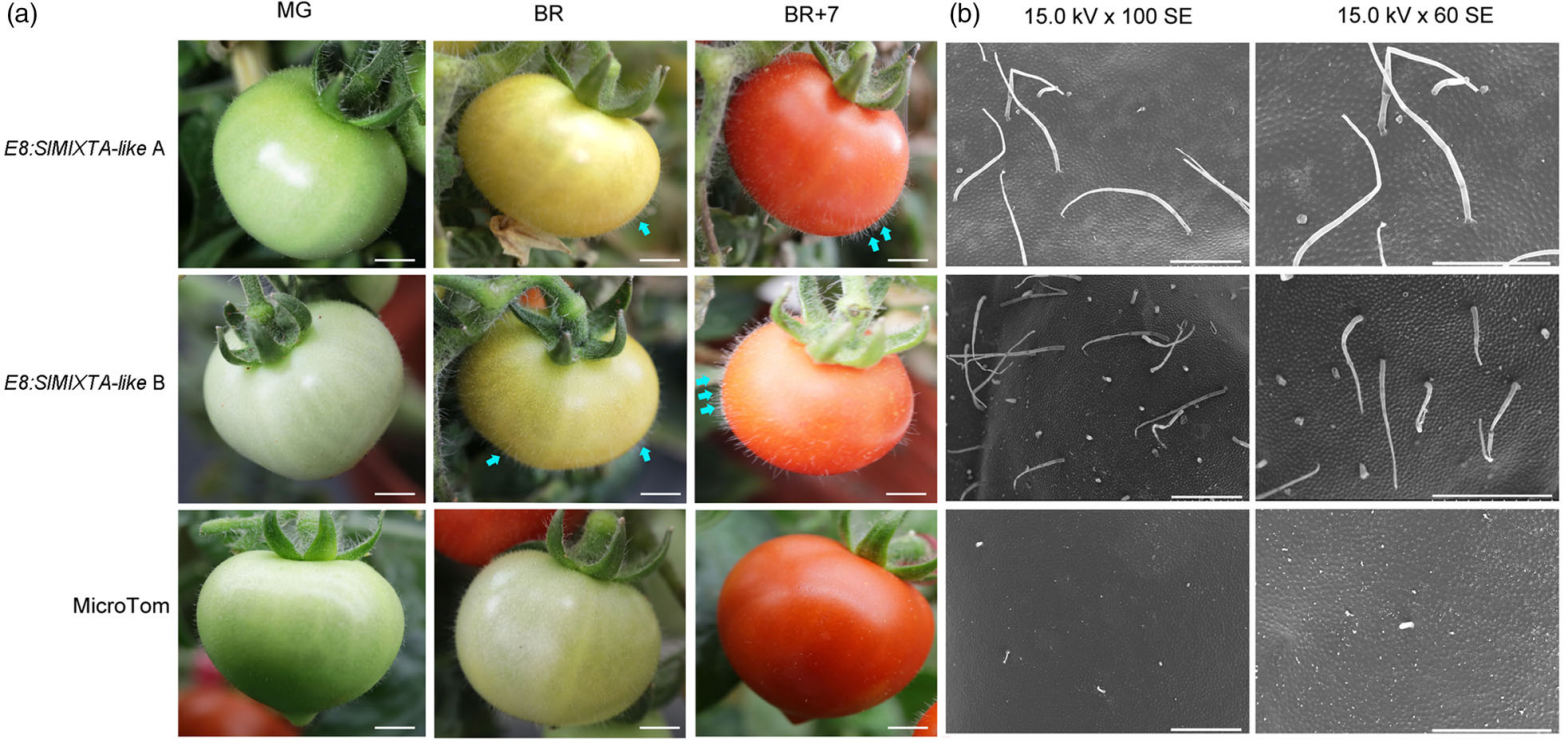
Fruit‐specific expression of *SlMIXTA‐like* enhances trichome formation on tomato fruit. (a) Phenotypes of transgenic and MicroTom tomato fruit at different stages. MG, mature green; BR, breaker; BR+7, 7 days post breaker. Scale bars show 4 mm. (b) Scanning electron microscope observation of trichome structures on the surface of T1 generation transgenic and MicroTom tomato fruit. Samples were analysed at BR+7. Scale bars show 500 μm.

To better characterize the potential function of *SlMIXTA‐like* in tomato fruit, we then overexpressed *SlMIXTA‐like* under fruit‐specific *E8* promoter (see Experimental procedures). A total of 14 T0 lines were obtained and preliminary screening indicated 13 of them had significantly higher *SlMIXTA‐like* expression in ripe fruit compared to MicroTom (Figure S6). High expression level was found in the fruit of lines A and B, and these lines were chosen to grow to T1 generation for further investigation (Figure S6). Compared to MicroTom plants, both *E8:SlMIXTA‐like‐*A and *E8:SlMIXTA‐like‐*B have no significant difference by mature green (MG) stage. However, at MG, as the induction of *E8* promoter (Deikman, 1996; Fischer, 1988), *SlMIXTA‐like* begins to express and both transgenic lines begin to accumulate trichomes (Figure 3a and Figure S7). Further investigation using scanning electron microscopy (SEM) confirmed that the *E8:SlMIXTA‐like* fruit produces higher density of trichome on fruit surface (Figure 3b). Compared to *35S:SlMIXTA‐like* plant, the fruit of *E8:SlMIXTA‐like* transgenic lines has significantly higher expression of *SlMIXTA‐like* (Figure 2b and Figure S6). Thus, the trichome density on the surface of latter is significantly increased (Figure 3). This matches previous conclusion that *SlMIXTA‐like* is associated with trichome formation in tomato (Ewas *et al*., 2016; Lashbrooke *et al*., 2015).

In addition to trichomes, we also noticed significant changes in the colour of *E8:SlMIXTA‐like* ripe fruit. Compared to MicroTom and *35S:SlMIXTA‐like* fruit, *E8:SlMIXTA‐like* fruit shows orange colour, indicating changes in the contents of metabolites due to the overexpression of *SlMIXTA‐like* in fruit (Figures 2d and 3a). All of the results above indicate that the overexpression of *SlMIXTA‐like* in tomato fruit not only induces trichome formation on fruit surface, but also alters metabolic patterns in fruit.

### Ectopic expression of *SlMIXTA‐like* in tomato fruit alters primary metabolism

**Figure 4:**
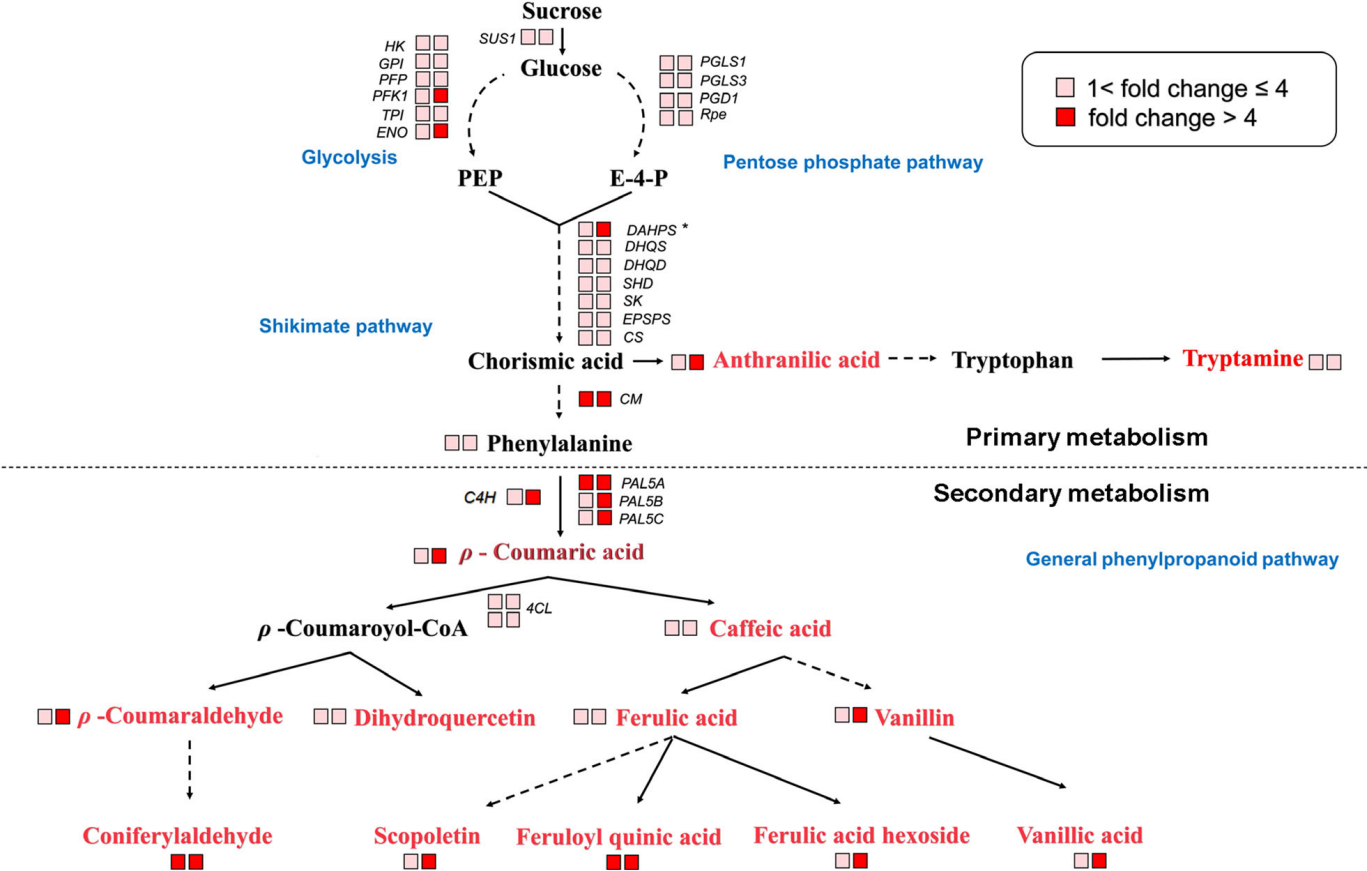
Overexpression of *SlMIXTA‐like* in tomato fruit significantly changes the dynamic of primary and secondary metabolism. Schematic representation of metabolic changes in tomato fruits expressing *SlMIXTA‐like*. Based on transcriptome and metabolomics data, genes and metabolites which are significantly increased in the transgenic fruits (line A and line B) are coloured. Data are represented as fold change compared to MicroTom. Detailed data are shown in Tables S2 and S3.

**Figure 5:**
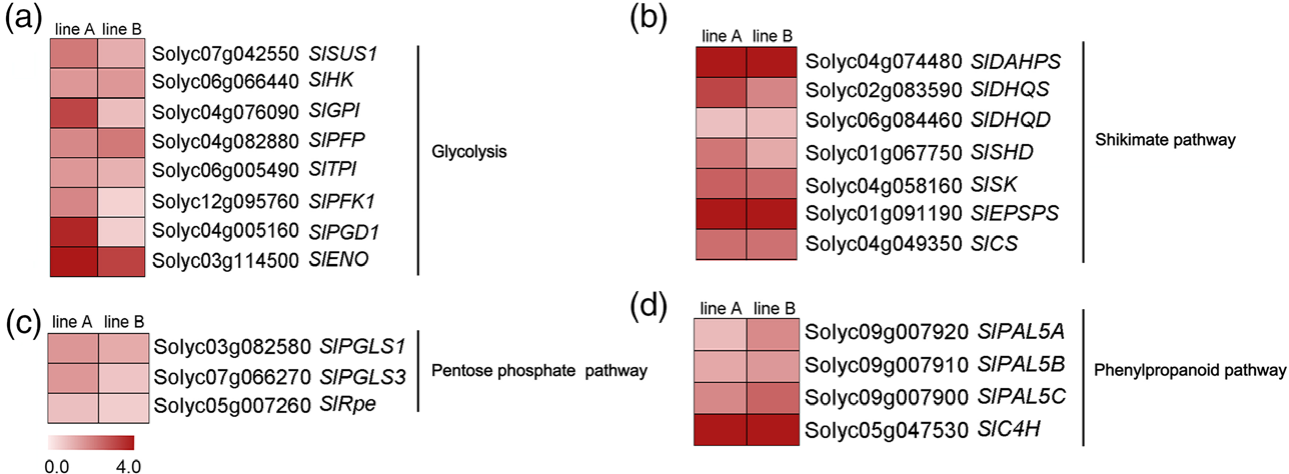
RT‐qPCR confirmation of differently expressed genes. Genes involved in glycolysis (a), pentose phosphate pathway (b), shikimate pathway (c) and phenylpropanoid pathway (d) are analysed. Data are represented in fold changes compared to MicroTom in log2 scale for both lines A and B. Gene IDs and abbreviations are explained in Table S4.

To better investigate the potential roles of *SlMIXTA‐like* in metabolic regulation, we performed both transcriptome and metabolic profiling for both *E8:SlMIXTA‐like*‐A and *E8:SlMIXTA‐like*‐B fruit (Data S2). Compared to MicroTom, there are 1696 differently expressed genes (DEGs) (fold change ≥ 1.5, FDR < 0.05) in the fruit of *E8:SlMIXTA‐like* line A and 2670 DEGs in line B. In total, 948 differently expressed genes are shared for both lines (Figure S8a). Among the shared DEGs, we found many genes involved in glycolysis, and pentose phosphate pathway and shikimate pathway are up‐regulated in both *E8:SlMIXTA‐like* fruits (Figure 4, Figure S8b and Table S2). We further checked the changes in gene expression levels by RT‐qPCR and confirmed genes involved in glycolysis (*SlSUS1*,* SlHK, SlGPI, SlPFP, SlPFK1, SlTPI, SlENO*), pentose phosphate pathway (*SlPGLS, SlPGD1* and *SlRpe*) and shikimate pathway (*SlDAHPS, SlDHQS*,* SlDHQD*,* SlSHD*,* SlSK*,* SlEPSPS*,* SlCS*,* SlCM*) are significantly induced in the fruit of both *E8:SlMIXTA‐like* lines (Figure 5a–c). In addition to that, downstream general phenylpropanoid pathway genes (*PAL5A*,* PAL5B* and *PAL5C*) are also up‐regulated (Figure 5d). To exclude the influence of trichomes’ presence to the expression of primary metabolic genes, we removed the trichomes of *E8:SlMIXTA‐like*‐B fruit and measured gene expression pattern in the pericarp (Figure S9a). Still, we can see the induction of key primary metabolic genes is directly associated with *SlMIXTA‐like* overexpression (Figure S9b). All these results indicate overexpression of *SlMIXTA‐like* in tomato fruit reprogrammes primary metabolism, by inducing the expression of genes involved in glycolysis, pentose phosphate pathway and shikimate pathway.

In order to confirm how fruit‐specific expression of *SlMIXTA‐like* alters primary metabolism, we performed widely targeted metabolomics analysis to MicroTom and *E8:SlMIXTA‐like* tomato fruit at ripe stage (7 days after breaker). Compared to MicroTom fruit, there are 208 metabolites significantly changed in *E8:SlMIXTA‐like*‐A, while for *E8:SlMIXTA‐like*‐B the number is 237. In total, 109 metabolites are significantly changed in both *E8:SlMIXTA‐like* lines (Figure S10a and Data S3). In addition to previously reported compounds in lipid biosynthesis (Lashbrooke *et al*., 2015), there is also significant increase in aromatic amino acid derivatives and general phenylpropanoids (Figure 4a, Figure S10b–c and Table S3). We further confirmed these using UPLC and found phenylpropanoid compounds such as ρ‐coumaric acid, chlorogenic acid and ferulic acid are significantly enriched in *E8:SlMIXTA‐like,* fruit while rutin, a major flavonol, is not changed (Figure S11a–d). Noticeably, ρ‐coumaric acid is the compound we used to perform mGWAS analysis (Figure 1a, b) and these data further attest *SlMIXTA‐like* is the gene responsible for the regulation of ρ‐coumaric acid content in tomato.

As fruit of both *E8:SlMIXTA‐like* lines A and B showed orange colour (Figure 3), we also checked the expression of carotenoid biosynthetic genes and the contents of major carotenoids in both MicroTom and transgenic fruit. Compared to MicroTom, we saw significant induction of carotenoid biosynthesis genes in *E8:SlMIXTA‐like* fruit (Figure S12a). Metabolic analysis also indicates there are increased contents of lycopene and β‐carotene (Figure S12b). This matches previous observation on overexpression of *SlMX1* in Ailsa Craig tomato (Ewas *et al*., 2016). All these data above indicate instead of the inhibition of carotenoid biosynthesis, and the orange colour of *E8:SlMIXTA‐like* fruit is mainly due to the accumulation of phenylpropanoid compounds.

Taken together, all these data indicate ectopic expression of *SlMIXTA‐like* in tomato fruit significantly changes transcriptional level of primary metabolic genes, resulting in the change in contents of related metabolites: the dynamics of primary metabolism has been significantly changed by overexpression of *SlMIXTA‐like* in tomato fruit.

### SlMIXTA‐like directly interacts with *SlDAHPS* to change the dynamics of primary metabolism

Previous study indicates AtMYB12, a TF from *Arabidopsis thaliana*, can directly binds to the promoter region of genes encoding enolase (*SlENO*) and 3‐deoxy‐d‐arabinoheptulosonate 7‐phosphate synthase (*SlDAHPS*). As a result, genes involved in glycolysis, pentose phosphate pathway and shikimate pathway are up‐regulated, redirecting carbon flux towards aromatic amino acid biosynthesis (Zhang *et al*., 2015). As similar changes were seen in *E8:SlMIXTA‐like* fruit, we predict SlMIXTA‐like uses a similar mechanism to control primary metabolism.

**Figure 6:**
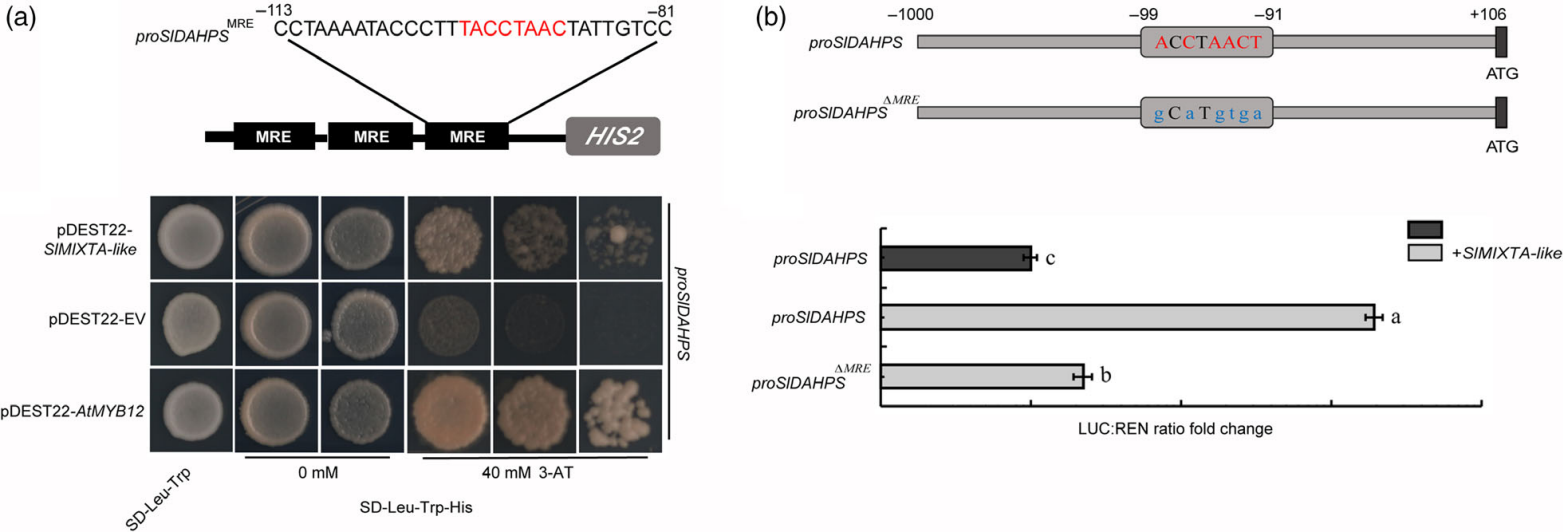
SlMIXTA‐like directly binds to the promoter of *SlDAHPS* to induce its expression. (a) Yeast one‐hybrid indicates SlMIXTA‐like directly interacts with the MRE in *SlDAHPS* promoter. The *SlDAHPS* promoter sequence from −113 to −81 containing the MRE motif was repeated three times and fused to the *HIS2* reporter gene in Y1H assay. AtMYB12 was used a positive control. (b) SlMIXTA‐like directly binds to MRE in *proSlDAHPS* to induce its expression. The *SlDAHPS* promoter sequence (−1000 to + 106) containing the MRE motif (from −99 to −91) was inserted into the reporting system. A mutated version (*∆* MRE) was designed as a negative control. Error bars show SEM (*n* = 3). Different letters indicate significantly different values at *P* < 0.05 (one‐way ANOVA, Tukey's *post hoc* test).

We first performed yeast one‐hybrid (Y1H) assay to test whether SlMIXTA‐like can directly bind to the promoter region of *SlENO* and *SlDAHPS*. Compared to AtMYB12, SlMIXTA‐like showed similar binding ability to *proSlDAHPS* (Figure 6a). For *proSlENO*, although direct binding activity was found for AtMYB12, no direct binding activity was shown for SlMIXTA‐like (Figure S13a). We further confirmed this by dual‐luciferase report system. SlMIXTA‐like can directly activate *SlDAHPS* promoter in *Nicotiana benthamiana* protoplast, whereas mutation of the MYB recognition element (MRE) in *SlDAHPS* promoter significantly reduces the activity (Figure 6b). For *proSlENO*, however, no significant induction by SlMIXTA‐like was observed (Figure S13b, c). To further confirm SlMIXTA‐like can directly bind to the promoter of *SlDAHPS*, we generated transgenic plants with *FLAG* tagged *SlMIXTA‐like* driven by *E8* promoter. The *E8:FLAG‐SlMIXTA‐like* fruit showed similar trichome enrichment, and chromatin immunoprecipitation (ChIP)‐qPCR shows significant enrichment of FLAG‐SlMIXTA‐like binding around the MRE of *proSlDAHPS* (Figure S14). All these data together indicate SlMIXTA‐like directly binds to the MRE in the promoter of *SlDAHPS* to induce its expression.

**Figure 7:**
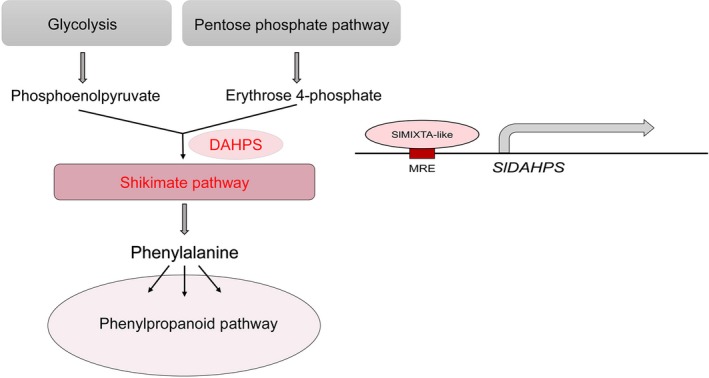
Proposed model for the molecular mechanism of SlMIXTA‐like mediated primary metabolic regulation in tomato. SlMIXTA‐like directly binds to *proSlDAHPS* to induce its expression. Enhanced DAHPS activities direct carbon flux towards shikimate pathway and downstream phenylpropanoid pathway.

3‐Deoxy‐d‐arabinoheptulosonate 7‐phosphate synthase is reported as rate‐limiting enzyme for shikimate pathway. And overexpression of *DAHPS* alone is enough to induce the expression of genes involved in shikimate pathway, resulting enhanced production of aromatic amino acids and downstream secondary metabolites (Tzin *et al*., 2012, 2013). SlMIXTA‐like can directly bind to the MRE in the promoter region of *SlDAHPS* and induce its expression. The induction of *SlDAHPS* significantly changes the dynamics of primary metabolism, redirecting carbon flux towards the synthesis of aromatic amino acid, providing substrate for downstream secondary metabolism (Zhang *et al*., 2015) (Figure 7).

In addition to regulating trichome formation, SlMIXTA‐like manipulates the dynamics of primary metabolism by directly inducing the transcriptional level of *SlDAHPS*. Through one TF, tomato can reprogramme primary metabolism for the production of specialized metabolites and form trichomes as storage compartments.

## Discussion

One of the biggest challenges for plant metabolic engineering is to identify transcription factors regulating key steps. Important metabolic pathways often have multiple levels of regulation to maintain its stability. Compared to structural genes, mutations in regulatory genes might not have significant phenotype due to the redundancy. Although there are examples of using natural mutants or mutagenesis approaches to identify TFs (Adato *et al*., 2009; Ballester *et al*., 2009; Borevitz *et al*., 2000; Schwinn *et al*., 2006), new high‐throughput methodologies are needed. The development of broad profiling technologies such as genome sequencing, transcriptome analysis and metabolomics profiling provides a perfect solution to this situation (Fang *et al*., 2019). Population genetic combined with omics can help us to detect minor content changes and link them to nucleic acid sequence difference at base pair level. As this forward genetics strategy is unbiased and accurate, it can provide more confident candidates for further study (Luo, 2015).

In plants, specialized metabolites are not synthesized in all cells. This is largely due to the variation of activities of TFs in different tissues. As a result, the barriers of cell types can be broken to produce specialized metabolites if right regulators can be identified (Fu *et al*., 2018). Recent studies reveal that glandular trichomes (GTs) of tomato contain large amount of secondary metabolites (Balcke *et al*., 2017; Glas *et al*., 2012; Huchelmann *et al*., 2017). In order to manage such significant change in secondary metabolism, the primary metabolism in trichome has been significantly altered (Balcke *et al*., 2017). As there are already reports about TFs regulating trichome formation (Glover *et al*., 1998; Lashbrooke *et al*., 2015; Perez‐Rodriguez *et al*., 2005), as well as TFs regulating primary metabolism (Zhang *et al*., 2015), we are wondering if any of them can directly regulate both trichome formation and primary metabolism. SlMIXTA‐like was reported as regulator for conical cell development and trichome formation (Ewas *et al*., 2016; Lashbrooke *et al*., 2015). In this study, our mGWAS analysis indicated that it is also a regulator for plant metabolism. We found SlMIXTA‐like can directly bind to the MRE in the promoter region of *SlDAHPS* to activate its expression. Overexpression of *SlDAHPS* can enhance the activities of shikimate pathway to reprogramme primary metabolism, redirecting carbon flux towards the production of aromatic amino acids and their derivatives, which are the major compounds found to be stored in trichomes. By using the same TF to control trichome formation and primary metabolic regulation, plants can coordinate the production of specialized compounds and the initiation of storage compartments. As a result, they can provide accurate regulation to the production and storage of toxic specialized metabolites without impairing normal growth.

Once a TF being identified, changing its expression pattern can break the production barriers between different tissues (Fu *et al*., 2018). However, constitutive expression of TFs in plants is shown to be less effective than tissue‐specific expression (Luo *et al*., 2008). This is likely due to the dramatic changes in primary and secondary metabolism attenuate normal plant growth (Zhang *et al*., 2015). When *SlMIXTA‐like* is driven by *35S* CaMV promoter, it maintains its regulatory function to trichome formation. However, its function on primary metabolism is attenuated by the plants and losses the significance (Ewas *et al*., 2016; Lashbrooke *et al*., 2015). Tomato fruit is a perfect chassis for metabolic engineering. This is largely due to the high content of primary metabolites (sugars, amino acids, etc.,). In addition, fruit is the reproductive organ of tomato, which is produced at the late stage of tomato growth cycle. Therefore, changing its primary metabolism has little effects on vegetative growth. When *SlMIXTA‐like* is expressed under fruit‐specific *E8* promoter, its regulatory function to primary metabolism is maximally released (Figures 2 and 3).

Previously, MYB12 was found to directly bind to the promoter region of key primary metabolic genes (*ENO* and *DAHPS*). Overexpression of *AtMYB12* in tomato fruit can guide the carbon flux towards the production of aromatic amino acids (Phe, Tyr, Trp). So, it can be used as a general tool for phenylpropanoid engineering. However, as MYB12 can also directly bind to the promoter of flavonol biosynthetic genes (*CHS*,* F3H, FLS*) (Hartmann *et al*., 2005; Zhang *et al*., 2015), *AtMYB12* overexpression tomato produces mainly flavonols (Luo *et al*., 2008; Zhang *et al*., 2015). In order to produce other phenylpropanoids, flavonol biosynthesis needs to be blocked by using natural mutants or other strategies (Zhang *et al*., 2015). SlMIXTA‐like, however, only controls the expression of shikimate pathway genes (*DAHPS*) (Figure 6). So, its induction of phenylpropanoid biosynthesis has no preference, making it a better general tool for metabolic engineering.

## Experimental procedure

### Genome‐wide association analysis

A total of 2 037 679 SNPs (MAF > 5% and Missing rate < 10%) for 351 accessions (Table S1) were used to perform the genome‐wide association analysis. Efficient Mixed‐Model Association eXpedited (EMMAX) was used to conduct all associations (Kang *et al*., 2010). GWAS analysis was conducted as described previously (Zhu *et al*., 2018). The genome‐wide significance thresholds of all the traits were set with a uniform threshold (*P *=* *1/*n*,* n* is the effective number of independent SNPs). The unified threshold (*P* = 2.87E10‐7) was used to filter the SNPs for all the metabolites. LD (linkage disequilibrium) analyses were performed based on all the SNPs (MAF > 0.05) using Haploview software. To reduce the redundancy of mGWAS signals, the lead SNP within a 1 Mb window for each metabolite was extracted as one signal.

### Plant material and growth conditions

*Solanum lycopersium* cv MicroTom seeds were purchased from PanAmerican Seed^tm^. Plants were grown in glasshouse under 16‐h light, 24°C and 60% humidity.

### Subcellular localization

Full‐length *SlMIXTA‐like* cDNA was amplified from pBin19‐*E8:SlMIXTA‐like* using primer pair *Sal*I‐SlMIXTA‐like‐F and SlMIXTA‐like‐*Kpn*I‐R and fused with GFP in the pSuper1300 (Llave *et al*., 2000;; Liu *et al*., 2017; Ni *et al*., 1995); the pSuper1300‐*SlMIXTA‐like* and pSuper1300 were individually transient injected into leaves of *Nicotiana benthamiana* by *Agrobacterium tumefaciens* strain GV3101 as described previously (Llave *et al*., 2000). Microscope inspection was done at 3 dpi (day past inoculation), and the peak wavelength of GFP was 488 nm.

### Plant transformation vectors’ construction and tomato transformation

To make pBin19‐*E8*‐GW plasmid, first, *proE8* was amplified from plasmid pSLJ.E8.1500 (Butelli *et al*., 2008) and inserted into pJIT60 to make pJIT60‐*proE8*. Gateway cassette was then inserted to make pJIT60‐*E8*‐GW. Finally, *E8*‐GW fragment was inserted into pBin19 to make pBin19‐*E8*‐GW. Full‐length CDS of *SlMIXTA‐like* (*Solyc02g088190*) was inserted into the destination vector through Gateway Cloning to make pBin19‐*E8:SlMIXTA‐like*. To make pBin19‐*35S:SlMIXTA‐like* plasmid, the full‐length CDS of *SlMIXTA‐like* was inserted into destination vector pBin19‐*35S*‐GW through Gateway Cloning. Tomato stable transformation was done by *Agrobacterium tumefaciens* strain EHA105 as previously described (McCormick *et al*., 1986).

### RNA extraction and RT‐qPCR

Both MicroTom and transgenic fruit were harvested at BR + 7. Fruit pericarp was ground into fine powder using liquid nitrogen. Total RNA samples were isolated from each sample using RNAiso Plus (Takara Bio, Kusatsu, Japan, AHF1820A) following the manufacturer's instruction. cDNA (complementary DNA) was synthesized from 1 μg of total RNA using a PrimeScript^tm^ RT reagent Kit with gDNA Eraser (Takara Bio, Kusatsu, Japan, AK4201). cDNA products were diluted into 2.5 ng/μL and used as templates for the qPCR. RT‐qPCR was performed using the Bio‐Rad CFX384. Each reaction (10 μL) consisted of 5 μL of iTaq^tm^ Universal SYBR Green Supermix (Bio‐Rad, Hercules, USA, #172‐5124), 1 μL each of forward and reverse primers and 3 μL of cDNA. Thirty‐nine cycles of amplification (pre‐incubation at 95°C for 2 min followed by each cycles consisting of 5 s at 95°C, 10 s at 60°C and added melting curve analysis during the 65–95°C). The results were calculated using Bio‐Rad CFX Manager software. With *SlUBI* as an internal control, the relative expression of each genes was calculated by the ∆∆Ct method (Schefe *et al*., 2006). The primer pairs for RT‐qPCR were designed using Primer3Plus (http://www.primer3plus.com) and blasted at NCBI database to ensure primer specific (see Table S4).

### Scanning electron microscopy (SEM) assay

For SEM, 2 mm^3^ of flesh tomato peel was collected at BR + 7 stage for MicroTom and transgenic tomato and fixed with 2% glutaraldehyde for 12 h. Samples were dried using critical point drying method. Scanning electron microscopy was performed using SU3500 at 15 kV (Panikashvili *et al*., 2009).

### Transcriptome and metabolic profiling

Both MicroTom and transgenic fruit were harvested at BR + 7. Fruit pericarp was ground into fine powder using liquid nitrogen. RNA isolation was performed using an RNeasy Plant Mini Kit (Qiagen, Stockach, Germany). The libraries were produced and sequenced by Illumina HiSeq2500/x. Raw sequences were filtered to remove the adaptor sequence, low‐quality reads (reads containing sequencing Ns > 10%) and short reads (Q < 10 nt), and the resulting sets of the high‐quality clean reads were used for transcriptome analysis. All clean reads were mapped to the reference genome using the TopHat (vision 2.1.1) (Kim *et al*., 2013) algorithm and conserved the mapped clean reads for the subsequent analysis. The mapped clean reads were calculated and then normalized to reads FPKM by Cuffquant and Cuffnorm software (vision 2.2.1). Differential expression analysis of two conditions/groups was performed using the DESeq2 R (vision 1.10.1) package, and DESeq2 (Anders and Huber, 2010) provides statistical routines for determining differential expression in digital gene expression data using a model based on the negative binomial distribution (Jiang and Wong, 2009) through indices of fold change (log_2_ ratio) and *P*‐value (false discovery rate, FDR). The resulting *P*‐values were adjusted using the Benjamini and Hochberg's approach for controlling the false discovery rate. Genes with an adjusted *P*‐value <0.01 and a log_2_ ratio >1.5 or <0.5 found by DESeq2 were assigned as differentially expressed. Clean data were deposited into the Genome Sequence Archive in Big Data Center, Beijing Institute of Genomics, Chinese Academy of Science, under accession number CRA001388 that are publicly accessible at http://bigd.big.ac.cn/gsa (Wang *et al*., 2017; Xu *et al*., 2018).

Metabolite profiling was carried out using a widely targeted metabolome method by Wuhan Metware Biotechnology Co., Ltd. (Wuhan, China) (http://www.metware.cn/). A liquid chromatography–electrospray ionization–tandem mass spectrometry (LC‐ESI‐MS/MS) system was used for the relative quantification of metabolites in dried tomato fruit samples (Zhu *et al*., 2018). The dried tomato fruit samples were crushed using a mixer mill (MM 400, Retsch) with a zirconia bead for 1.5 min at 30 Hz, and 100 mg dried powder was weighted and extracted overnight at 4°C with 1.0 mL pure methanol (or 70% aqueous methanol) containing 0.1 mg l^−1^ lidocaine (internal standard) for lipid‐solubility metabolites (or water‐solubility metabolites). Quantification of metabolites was carried out using a scheduled multiple reaction monitoring method (Chen *et al*., 2013).

### Compound extraction and UPLC measurement

Tomatoes were harvested at 7 days after breaker (BR + 7). Fruit pericarp was freeze‐dried and ground into fine powder. Extraction was performed as previously described (Zhang *et al*., 2015). Briefly, 100 mg of fruit powder was extracted with 5 mL 70% (v/v) MeOH for 12 h at 4°C, under agitation. After centrifugation at 3000 ***g*** at 4°C for 15 min, the supernatant was taken. The pellets were re‐extracted 5 mL 70% (v/v) MeOH at 4°C for another 2 h. After centrifugation, the supernatant was combined and further diluted five times with 70% MeOH. The combined solution was centrifugation at 18 000 ***g*** at 4°C for 15 min, and 10 μL supernatant was injected for before UPLC analysis.

For phenylpropanoid analysis, the samples were run on a Dionex Ultimate 3000 Series UPLC (Thermo Scientific, MA, USA). Separation was on a 100 × 2.1 mm 1.9 μm Hypersil Gold C18 column (Thermo Scientific, MA, USA) using following gradient of 0.1% formic acid in ultrapure water as mobile phase A and 100% acetonitrile as mobile phase B, run at 0.5 mL/min at 40°C: 0–0.5 min, 20% B; 0.5–5.5 min, 2%–25% B; 5.5–7 min, 25%–95% B; 7–7.5 min, 95% B; 7.5–7.6 min, 95%–2% B; and 7.6–12 min, 2% B. Detection was performed at 330 nm for chlorogenic acid, ρ‐coumaric acid and ferulic acid, 350 nm for rutin. All samples were performed in biological triplicate. Compounds were quantified using standards purchased from Sigma‐Aldrich (https://www.sigmaaldrich.com/).

### Yeast one‐hybrid assay

DNA fragment consisting of three copies of the *SlDAHPS* (3‐deoxy‐7‐phosphoheptulonate synthase, *Solyc04g074480*) and *SlENO* (Enolase, *Solyc03g114500*) promoter sequence containing the R2R3‐MYB core binding domain (−99 to −91 of *SlDAHPS* and −67 to −59 of *SlENO*) were chemically synthesized and then cloned into the pHis2‐Leu‐GW through Gateway Cloning (Zhang *et al*., 2015). The pHis2‐Leu‐*SlDAHPS* and pHis2‐Leu‐*SlENO* vectors were transformed into the yeast strain Y187 cells with the LiCl‐PEG method, respectively (Shim *et al*., 2013). The positive clones were selected on SD/‐Leu medium. Full‐length CDS of *SlMIXTA‐like* was introduced into pDEST22 vector to make the yeast expression vector pDEST22‐*SlMIXTA‐like* and transformed into yeast strains containing pHis2‐Leu‐*SlDAHPS* or pHis2‐Leu‐*SlENO*. The transformants were further grown on SD/‐Leu/‐Trp medium. Positive yeast clones were picked and grown in liquid culture and diluted into different concentrations (OD_600_ = 10^0^, 10^−2^, 10^−4^). Eight microlitres of suspension was spotted on the SD/‐Leu/‐Trp/‐His medium, with or without 3‐AT (0 or 30 mm). For comparison, full‐length cDNA of *AtMYB12* was used to replace *SlMIXTA‐like* as positive control (Zhang *et al*., 2015).

### Transient dual‐luciferase assays

The plasmid construction of dual‐luciferase assay was done by Golden Braid 2.0 cloning strategy (Sarrion‐Perdigones *et al*., 2013). Firstly, the 1 kb region promoter of *SlDAHPS* (−1000 to +106) and *SlENO* (−1021 to +150) was cloned and inserted into pUPD2 plasmid. The promoters were then inserted into GB_3‐α1 vector with *LUC* gene and *Tnos* to make *proSlDAHPS:LUC:Tnos* or *proSlENO:LUC:Tnos*. In the meantime, the *SlMIXTA‐like* gene under the control of a *35S* promoter was inserted into GB_3‐α2 vector to make *pro35S:SlMIXTA‐like:T35S*. The two α plasmids were combined by inserted into the Ω2 plasmid through new round of Golden Braid reaction. In the same construct, we combined the pGB3‐α1_ *pro35S:REN:Tnos* and pGB3‐α2_ *pro35S:P19:T35S* and converted to the Ω1 plasmid. Finally, the Ω1 and Ω2 plasmids were combined to make the α‐level plasmid (*pro35S:SlMIXTA‐like:T35S/proSlENO:LUC:Tnos/pro35S:REN:Tnos/pro35S:P19:Tnos* and *pro35S:SlMIXTA‐like:T35S/proSlDAHPS:LUC:Tnos/pro35S:REN:Tnos/pro35S:P19:Tnos*). The Ω‐level plasmid without the *pro35S:SlMIXTA‐like:T35S* part was used as the control (Sarrion‐Perdigones *et al*., 2013). The final binary vectors were directly transformed into *Agrobacterium tumefaciens* strain GV3101. The *Agrobacterium* cultures were grown to an OD_600_ of 0.8–1.0 and infiltrated into tobacco (*Nicotiana benthamiana*) leaves. Infiltrated leaves were harvested at 3dpi, and today protein was isolated by PBS solution. The transient expression was assayed using the Dual‐Luciferase Reporter Assay System (Promega, Madison, USA) (Llave *et al*., 2000). The ratio of LUC/REN was measured by a *Synergy* ^™^ H1 hybrid multimode microplate reader (BioTek) with according to the manufacturer's instructions.

### Chromatin immunoprecipitation (ChIP)

The N‐terminal of SlMIXTA‐like was fused with the FLAG‐tag (DYKDDDK) to generate *FLAG‐SlMIXTA‐like* vector and constructed pBin19‐*E8:FLAG‐SlMIXTA‐like* through Gateway Cloning. ChIP analysis was performed as previously described (Zhang *et al*., 2015).

ChIP‐qPCR was performed on three independent replicates with appropriate primers. The *ACTIN* gene from tomato was used as the internal control for the ChIP‐qPCR experiments. Data were represented as the ratio of target genes/*ACTIN* in ChIPed DNA to target genes/*ACTIN* in input DNA.

### Statistics

Unpaired, two‐tailed Student's *t*‐tests were used for comparison of individual lines with their relevant controls; *P* < 0.05 were considered significant. To compare measurements of multiple experiment designs with each other, we performed univariate ANOVA followed by the post hoc Tukey test of multiple pairwise comparisons to determine group differences; *P* < 0.05 were recognized as significant.

## Conflicts of interests

All authors declare no competing interests.

## Author contributions

YZ, SCW and JL designed the experiments. SY and MS performed the experiments, with help of YW, LZ, RF, YL and HG. SY, MS, SCW and YZ analysed the data and wrote the manuscript.

## Supporting information


**Figure S1** The effect of different alleles on the content of ρ‐coumaric acid (SlFM0124).
**Figure S2** Phylogenetic tree of SlMIXTA‐like protein and R2R3‐MYB protein family from *Arabidopsis thaliana*.
**Figure S3** Molecular characterization of SlMIXTA‐like.
**Figure S4** *SlMIXTA‐like* expression pattern in tomato.
**Figure S5** Overexpression of *SlMIXTA‐like* in tomato seedlings enhances trichome formation on stems.
**Figure S6** Screening of *SlMIXTA‐like* expression in T0 *E8:SlMIXTA‐like* tomato fruits.
**Figure S7** The expression of *SlMIXTA‐like* at different fruit development stages for both MicroTom and *E8:SlMIXTA‐like*‐B fruit.
**Figure S8** The analysis of RNA‐seq data in *E8:SlMIXTA‐like* line A and B.
**Figure S9** SlMIXTA‐like changes the expression of primary metabolic genes in the pericarp of *E8:SlMIXTA‐like* fruit.
**Figure S10** The analysis of metabolism data in *E8:SlMIXTA‐like* line A and B.
**Figure S11** The contents of rutin (a), ρ*‐*coumaric acid (b), chlorogenic acid (c) and ferulic acid (d) in *E8:SlMIXTA‐like*‐B and MicroTom.
**Figure S12** Expression of carotenoid pathway genes (a) and carotenoid contents (b) in *E8:SlMIXTA‐like‐*B and MicroTom fruit.
**Figure S13** SlMIXTA‐like can't directly bind to the promoter of *SlENO*.
**Figure S14** ChIP‐qPCR indicates SlMIXTA‐like directly binds to the promoter of *SlDAHPS in vivo*.

**Table S1** The list of collected 351 tomato varieties.

**Table S2** The list of *E8:SlMIXTA‐like*‐A and B common DEGs in RNA‐seq.

**Table S3** The list of SlMIXTA‐like‐A and B common DEMs in metabolic profiling.

**Table S4** The list of oligonucleotids used in this study.

**Data S1** The list of total 53 SNPs significantly associated with ρ‐coumaric acid in this study.

**Data S2** All sample RNA‐seq fpkm.

**Data S3** All sample metabolomics data.
