# Supplementary material for: Trichome regulator SlMIXTA‐like directly manipulates primary metabolism in tomato fruit
Source: Plant Biotechnol J. 2019 Jul 24;18(2):354–63. doi: 10.1111/pbi.13202 (PMC6953195; doi:10.1111/pbi.13202)
Supplement: Supplementary file 1 — Figure S1 The effect of different alleles on the content of ρ‐coumaric acid (SlFM0124). Figure S2 Phylogenetic tree of SlMIXTA‐like protein and R2R3‐MYB protein family from Arabidopsis thaliana. Figure S3 Molecular characterization of SlMIXTA‐like. Figure S4 SlMIXTA‐like expression pattern in tomato. Figure S5 Overexpression of SlMIXTA‐like in tomato seedlings enhances trichome formation on stems. Figure S6 Screening of SlMIXTA‐like expression in T0 E8:SlMIXTA‐like tomato fruits. Figure S7 The expression of SlMIXTA‐like at different fruit development stages for both MicroTom and E8:SlMIXTA‐like‐B fruit. Figure S8 The analysis of RNA‐seq data in E8:SlMIXTA‐like line A and B. Figure S9 SlMIXTA‐like changes the expression of primary metabolic genes in the pericarp of E8:SlMIXTA‐like fruit. Figure S10 The analysis of metabolism data in E8:SlMIXTA‐like line A and B. Figure S11 The contents of rutin (a), ρ‐coumaric acid (b), chlorogenic acid (c) and ferulic acid (d) in E8:SlMIXTA‐like‐B and MicroTom. Figure S12 Expression of carotenoid pathway genes (a) and carotenoid contents (b) in E8:SlMIXTA‐like‐B and MicroTom fruit. Figure S13 SlMIXTA‐like can't directly bind to the promoter of SlENO. Figure S14 ChIP‐qPCR indicates SlMIXTA‐like directly binds to the promoter of SlDAHPS in vivo. [file PBI-18-354-s005.pdf]

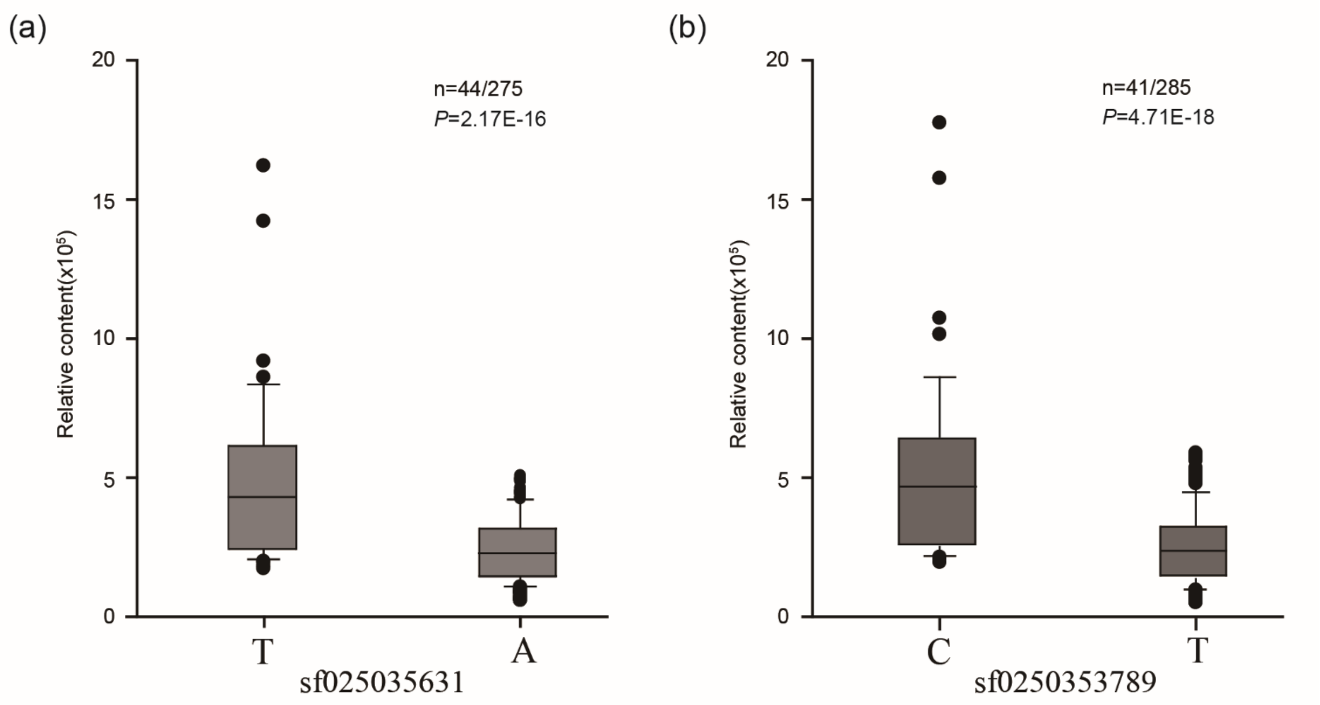

Figure S1. The effect of different alleles on the content of ρ-coumaric acid (SlFM0124). Boxplot (the middle lines indicate the median, the boxes show the range of the 5-95% of the total data, the whiskers indicate the interquartile range, and the outer dots are outliers) for ρ-coumaric acid (SlFM0124) content, plotted as a function of genotypes at SNP sf0250353631 (a) and sf0250353789 (b).

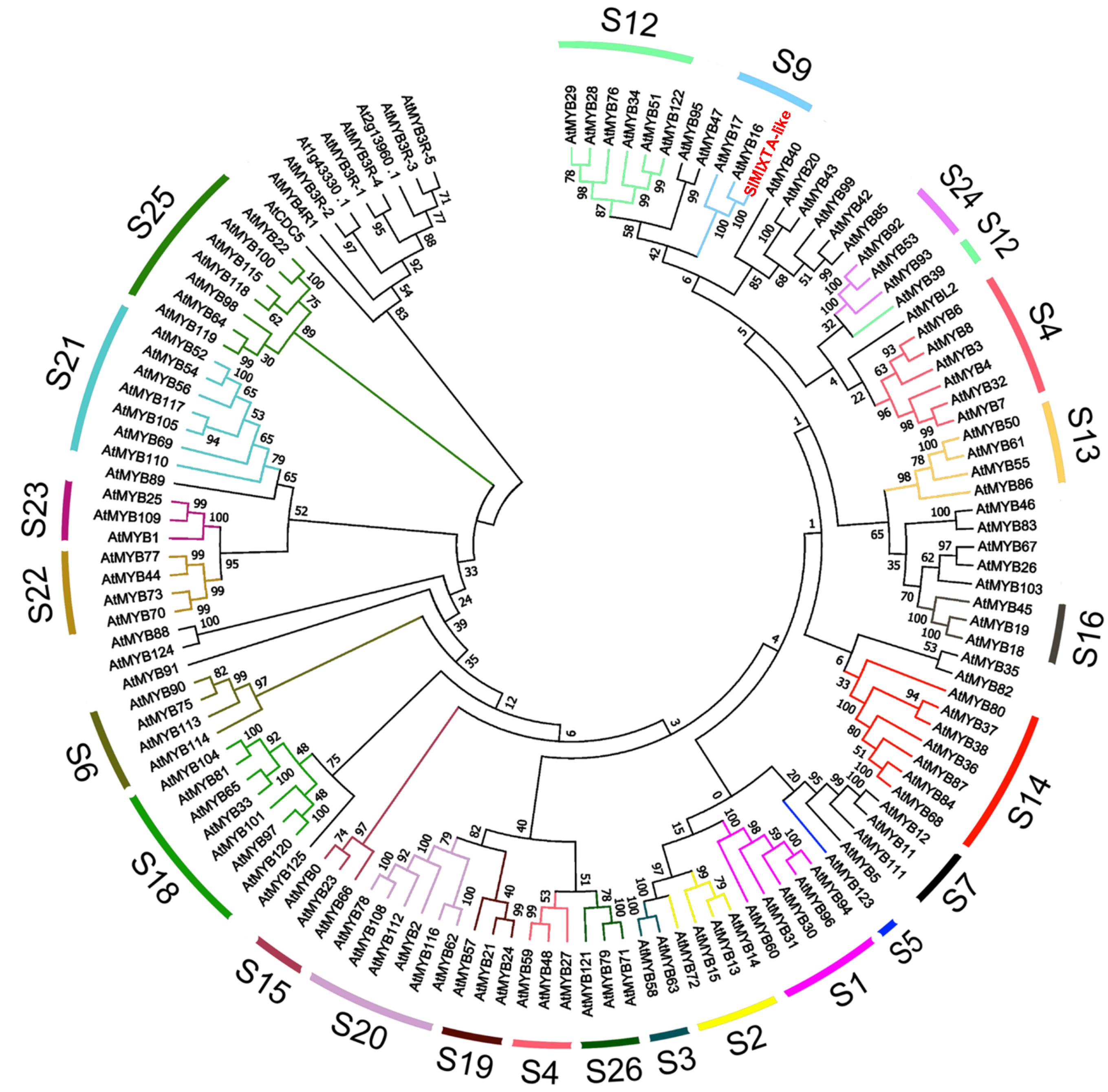

Figure S2. Phylogenetic tree of SlMIXTA-like protein and R2R3-MYB protein family from *Arabidopsis thaliana*. All the R2R3-MYB protein were download from the IT3F database (http://jicbio.bbsrc.ac.uk/IT3F/). Protein sequences were aligned using MEGA7.0 and evolutionary relationships were determined using Neighbor-Joining analysis with 1000 bootstrap replicates.

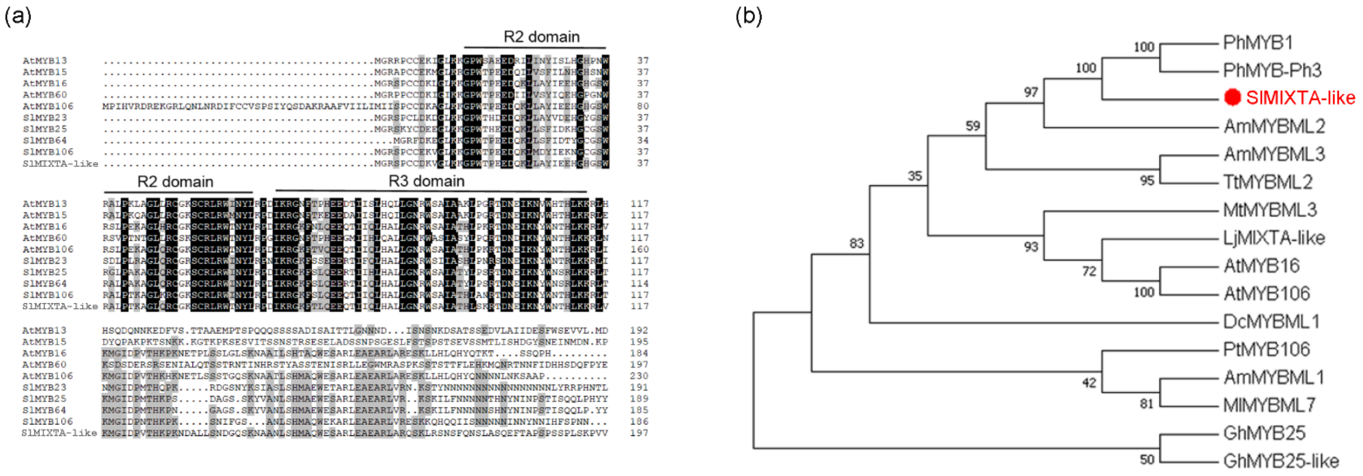

Figure S3. Molecular characterization of SlMIXTA-like. (a) Protein sequence alignment of partial *Arabidopsis thaliana* R2R3 MYB transcription factors and tomatoes. The R2R3 repeats of the DNA binding domain are annotated. (b) Phylogenetic tree of the MIXTA/MIXTA-like protein in different plants. The following protein sequences were used for the analysis: PhMYB1 (CAA78386.1), AtMYB16 (AT5G15310), AtMYB106 (AT3G01140), PhMYB-Ph3(CAA78386.1), AmMYBML2 (AAV70655.1), LjMIXTA-like (AEO27486.1), MtMYBML3 (XM_003621222.2), GhMYB25-like (ADZ55318.1), AmMYBML3 (AAU13905.1), TtMYBML2 (ACN69972.1), MlMYBML7 (AGO03571.1), PtMYB106 (XP_002311313.2), AmMYBML1 (CAB43399.1), GhMYB25 (AAK19616.1), MlMYBML7 (AGO03571.1).

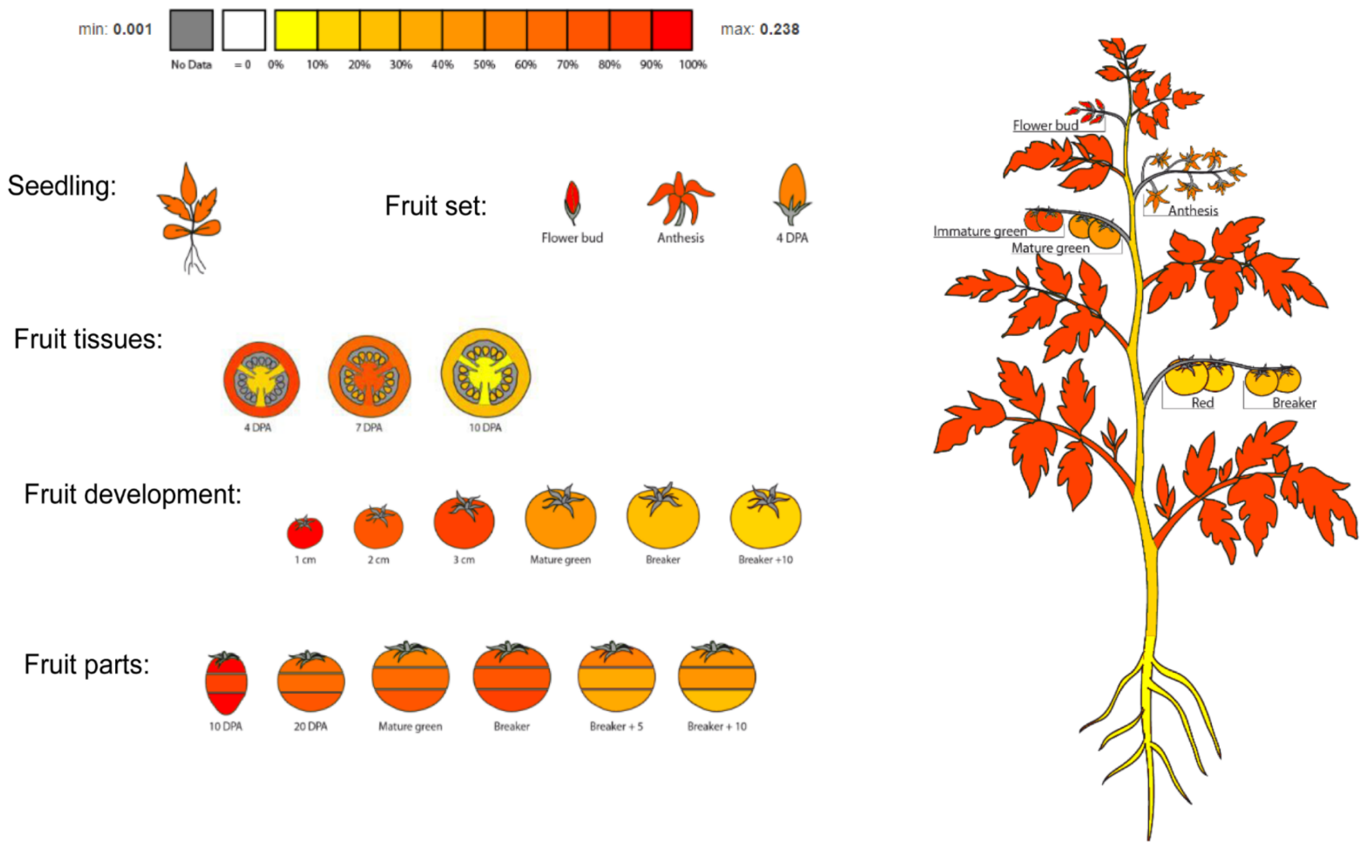

Figure S4. *SlMIXTA-like* expression pattern in tomato. Data was generated in TomExpress website (http://gbf.toulouse.inra.fr/tomexpress/www/welcome TomExpress.php)

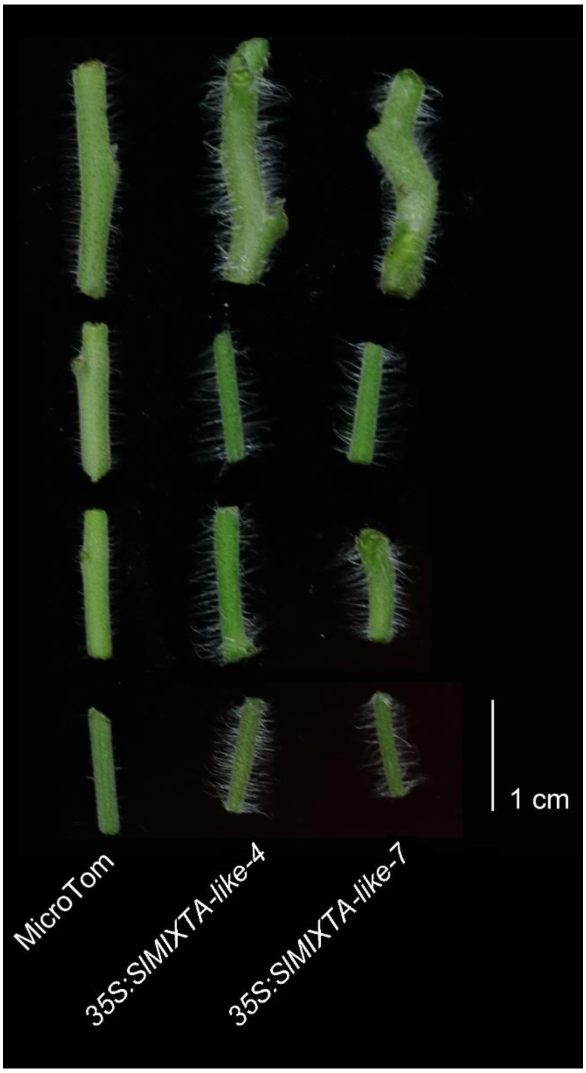

Figure S5. Overexpression of *SlMIXTA-like* in tomato seedlings enhances trichome formation on stems. Both MicroTom and *35S:SlMIXTA-like* stems were visual inspected when first flower opened. Stems on the same row were harvest at comparable part of the seedlings.

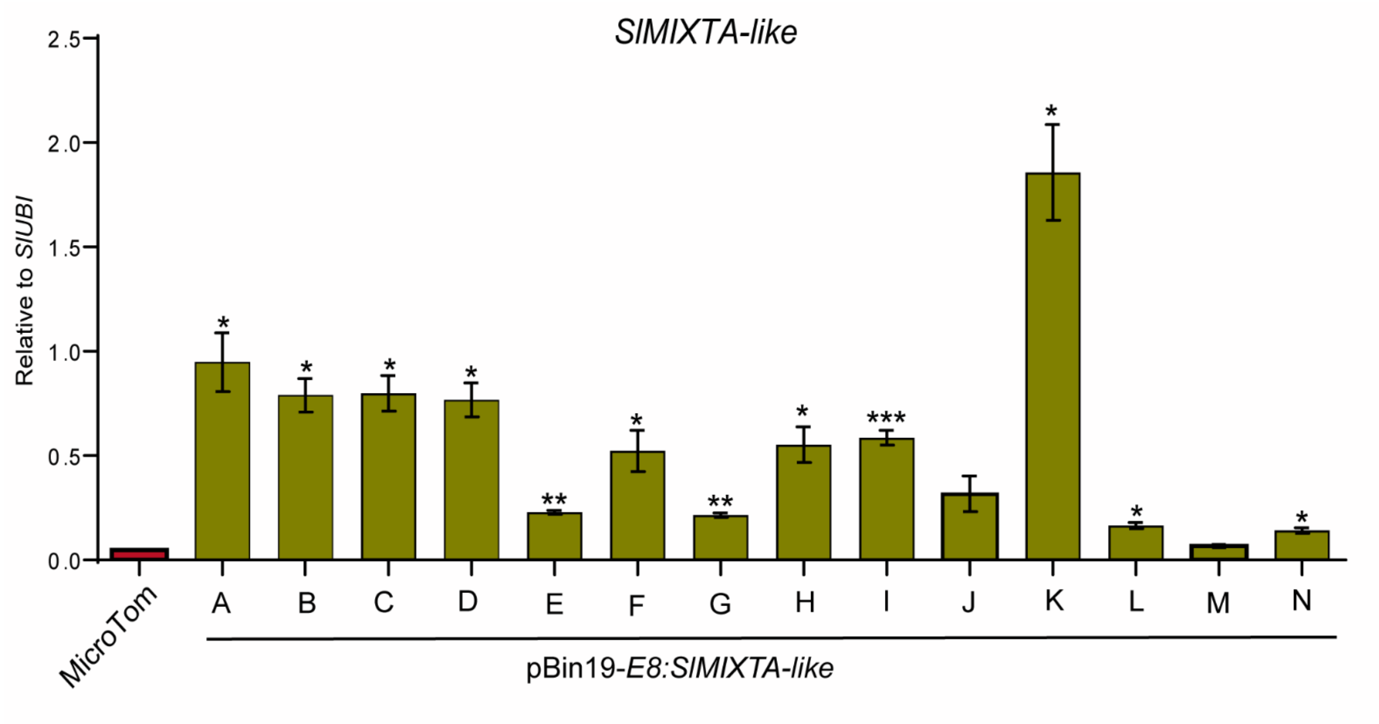

Figure S6. Initial screening of *SlMIXTA-like* expression in T0 pBin19-*E8:SlMIXTA-like* tomato fruits. *SlMIXTA-like* expression in line A to N of T0 transgenic and MicroTom tomato fruits were measured by RT-qPCR. Error bars represent the S.E.M of triplicates (three independent fruit at breaker + 3days). *, $p<0.05$. **, $p<0.01$. ***, $p<0.001$ (Student's t-test). Line K fails to produce any seeds.

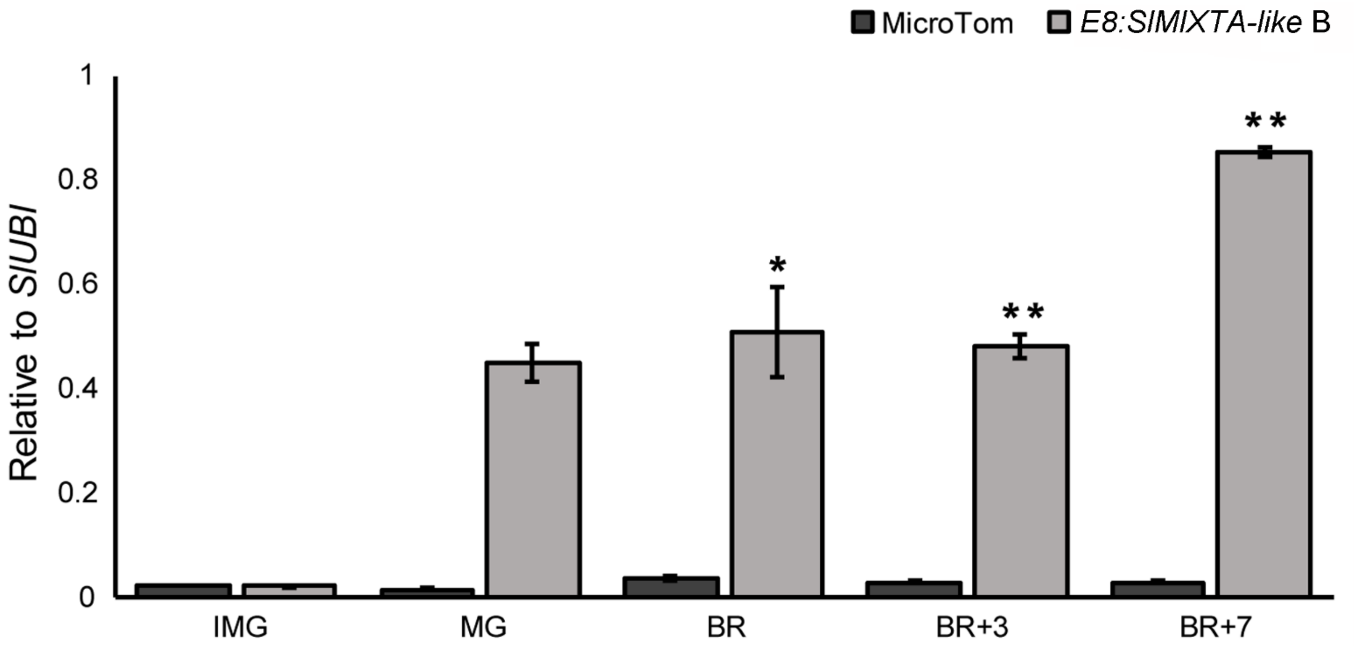

Figure S7. The expression of *SlMIXTA-like* at different fruit development stages for both MicroTom and pBin19-*E8:SlMIXTA-like* B fruit. Data were represented as mean ±S.E.M (n=3). *, $p<0.05$ (Student's t-test).

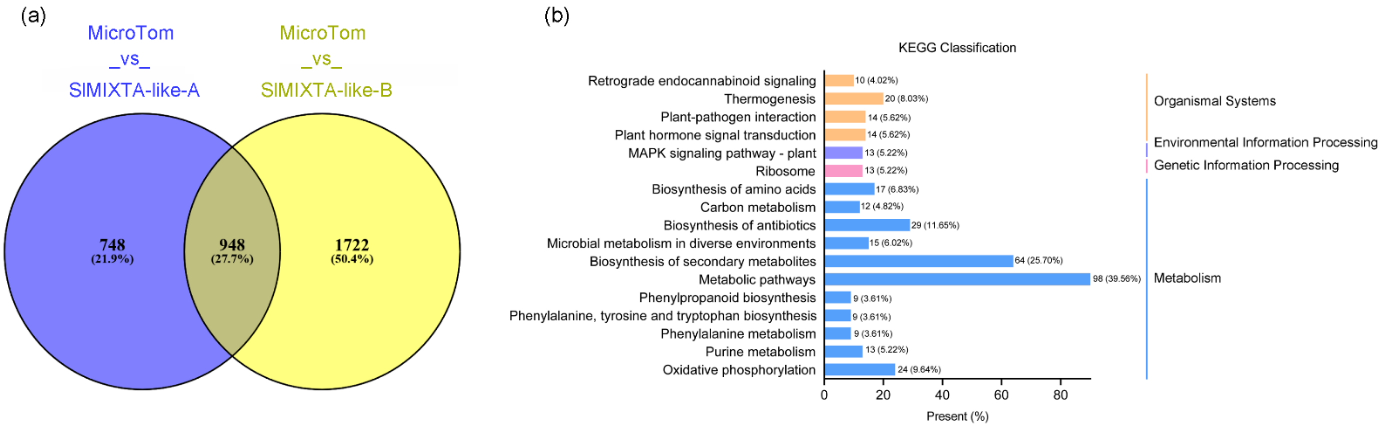

Figure S8. Transcriptome data for *E8:SlMIXTA-like* tomato fruit. (a) The venn map of differently expressed genes in *E8:SlMIXTA-like* line A and B using the Venny 2.1. The number represent the DEGs. (b) The KEGG classification of common DEGs in line A and B.

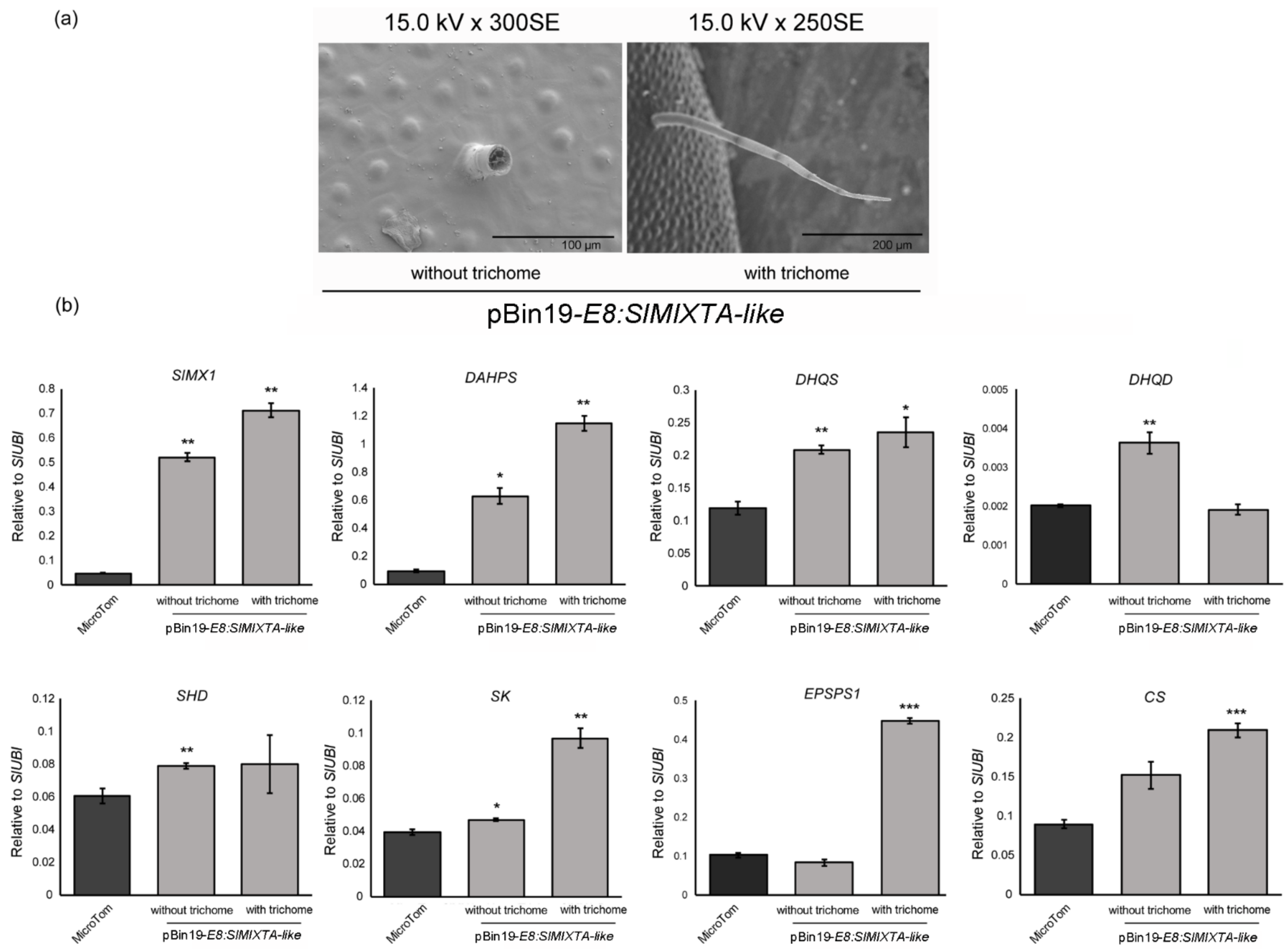

Figure S9. SlMIXTA-like changes the expression of primary metabolic genes in the pericarp of *E8:SlMIXTA-like* fruit. (a) SEM of *E8:SlMIXTA-like* B fruit surface after (left) and before (right) removing trichomes. Samples were treated and scanned at 3dpb. (b) Expression of *SlMIXTA-like* and key shikimate pathway genes in the fruit of MicroTom and *E8:SlMIXTA-like* B fruit with(out) trichomes. Samples were harvested at 3dpb. Data were represented as mean ± S.E.M (n=3). *, $p<0.05$ (Student's t-test).

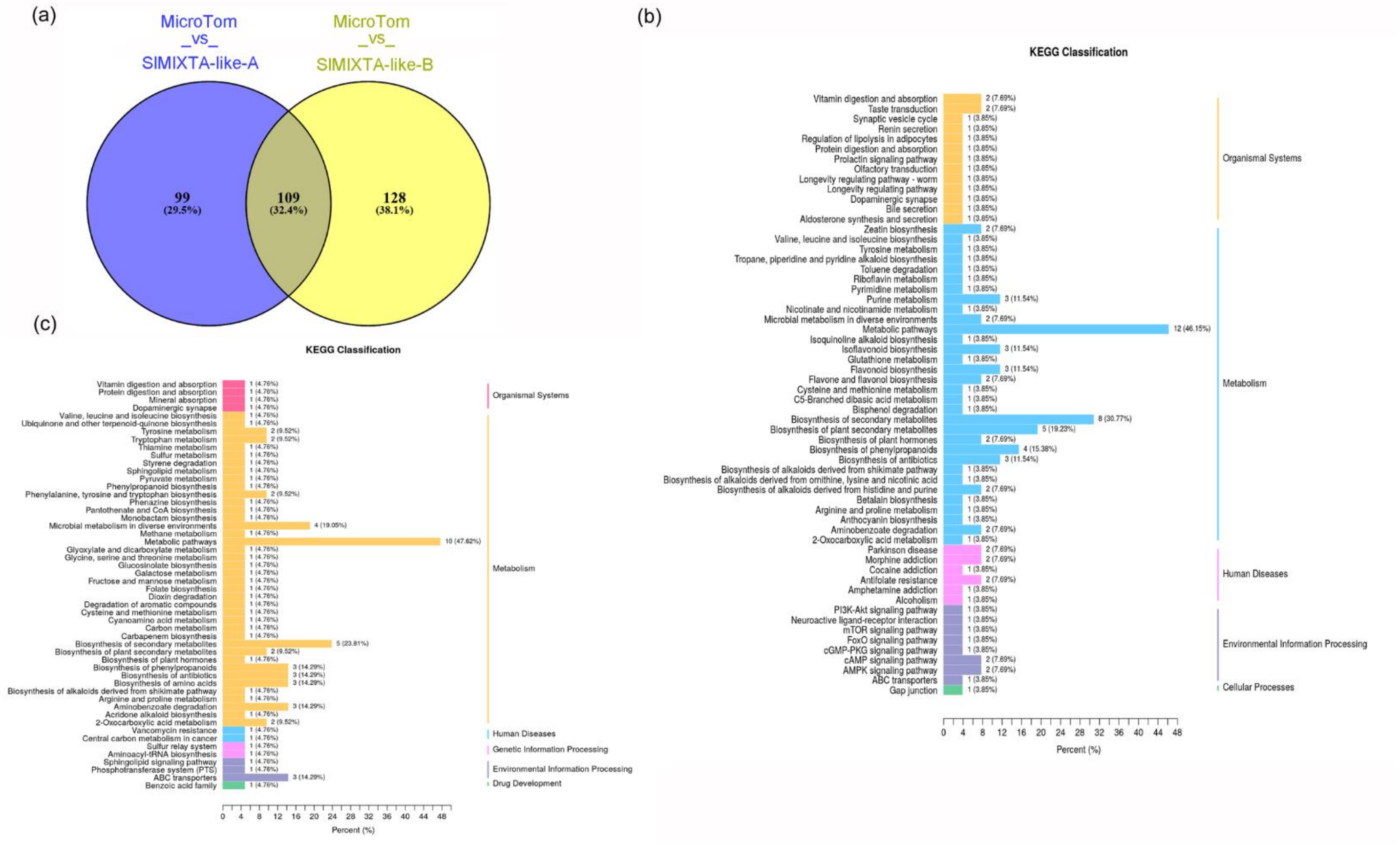

Figure S10. Metabolic profiling for *E8:SlMIXTA-like* tomato fruit (a) The venn map of differently accumulated metabolites *E8:SlMIXTA-like* line A and B using the Venny 2.1. The number represent the difference compounds. (b) The KEGG classification bar graph for metabolites up-regulated in both line A and B. (c) The KEGG classification for metabolites down-regulated in both line A and B.

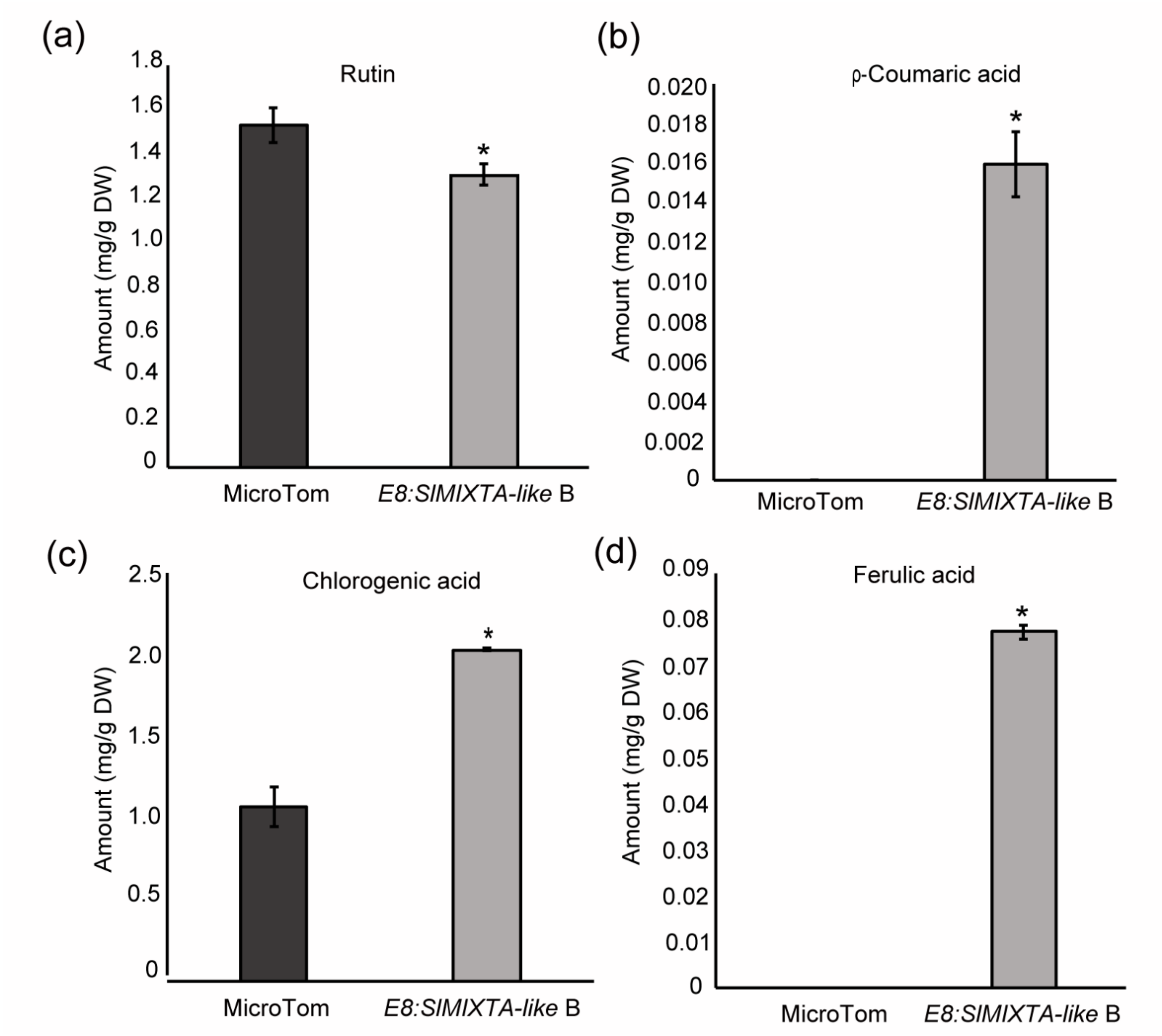

Figure S11. The contents of rutin (a), *ρ*-coumaric acid (b), chlorogenic acid (c) and ferulic acid (d) in *E8:SlMIXTA-like* B and MicroTom. Data were represented as mean ± S.E.M (n=3). *, $p<0.05$ (Student's t-test).

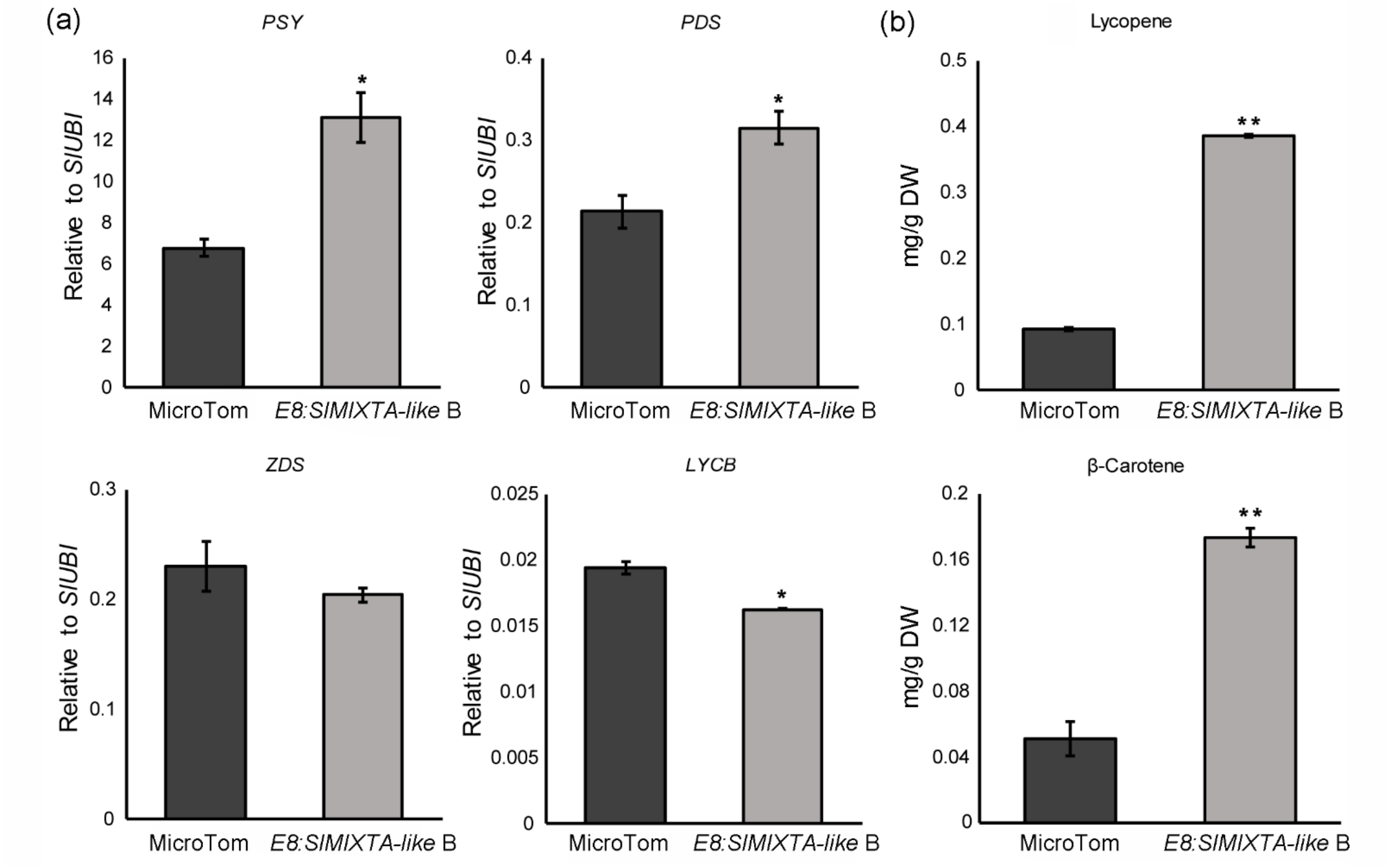

Figure S12. Expression of carotenoid pathway genes (a) and carotenoid contents (b) in *E8:SlMIXTA-like* B and MicroTom fruit. Samples were harvested at 7dpb. Data were represented as mean ± S.E.M (n=3). *, $p<0.05$ (Student's t-test).

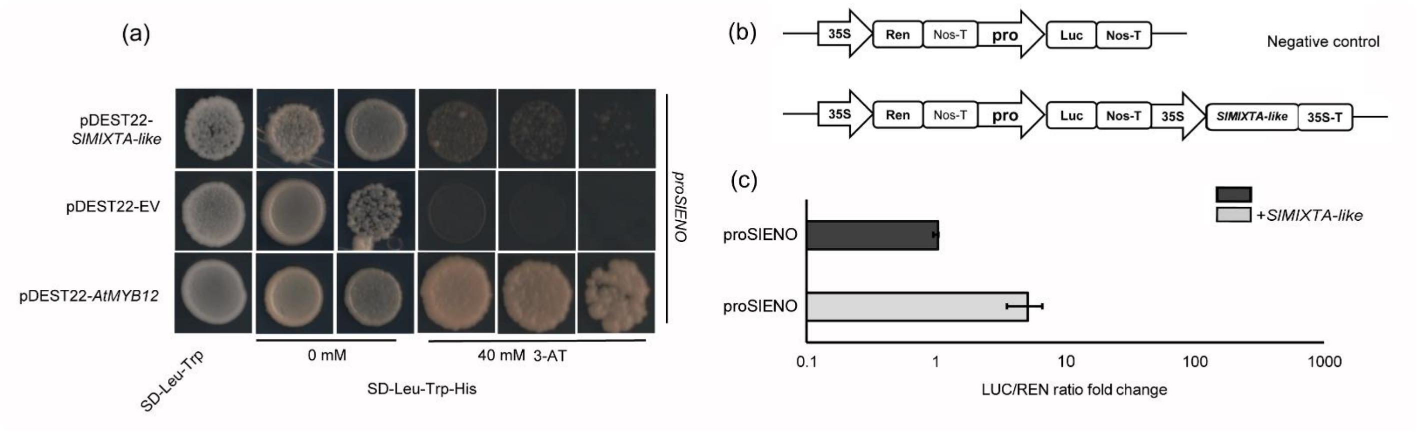

Figure S13. SlMIXTA-like can't directly bind to the promoter of *SlENO*. (a) Yeast one-hybrid indicates SlMIXTA-like cannot directly interact with *SlENO* promoter. AtMYB12 was used a positive control. (b) Schematic representation of fused vector and negative control vector in Dual-LUC assay. (c) Dual-luciferase assay indicates SlMIXTA-like can't bind to the promoter region of *SlENO* to induce its expression. Error bars show S.E.M (n=3). No significant difference ($P<0.05$) was observed.

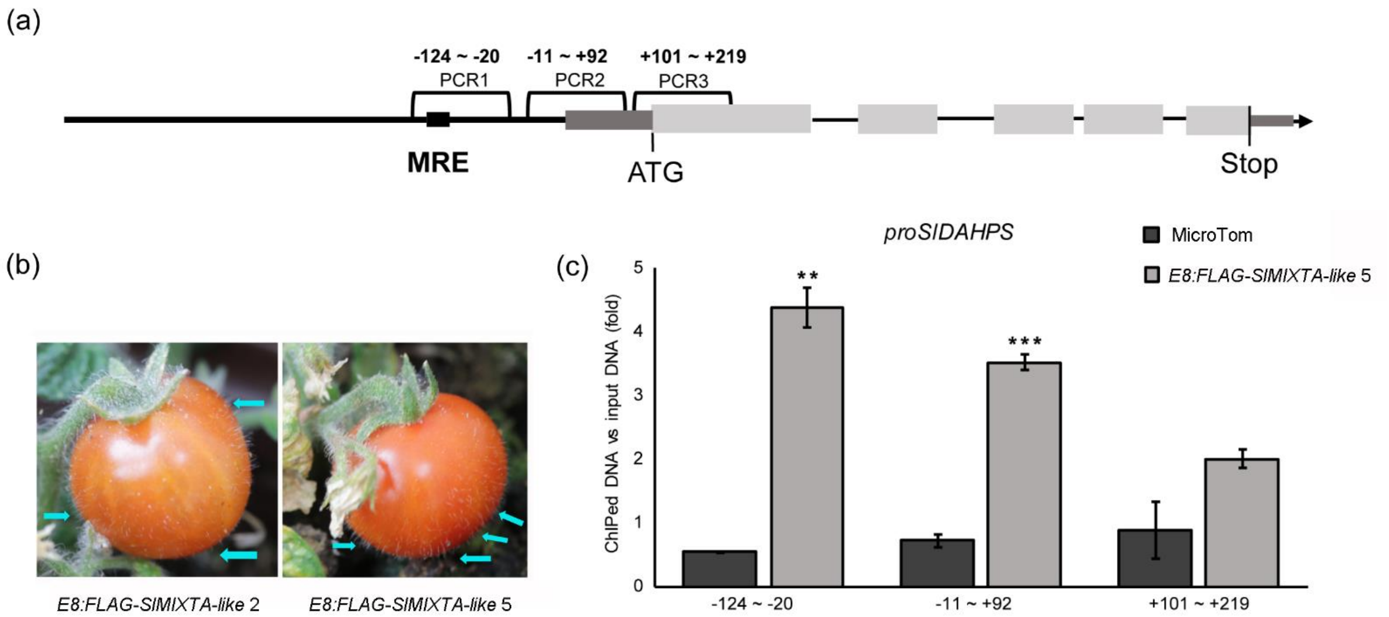

Figure S14. ChIP-qPCR indicates SlMIXTA-like directly binds to the promoter of *SlDAHPS in vivo*. (a) schematic representation of *SlDAHPS* gene structure and PCR primers design. Black box indicates MYB recognition site (MRE). (b) *E8:FLAG-SlMIATA-like* fruit show enhanced trichome density on ripe fruit. (c) Binding of SlMIATA-like to the *proSlDAHPS* was confirmed by ChIP-qPCR. Fruit were analyzed at 5dpb. The numbers on the horizontal axis below the bars correspond to the left and right borders of the amplified regions relative to the initial transcription start site. Data were represented as mean ± S.E.M (n=3). *, p<0.05 (Student's t-test).
